# Comparison of DNA extraction methods for 16S rRNA gene sequencing in the analysis of the human gut microbiome

**DOI:** 10.1038/s41598-023-33959-6

**Published:** 2023-06-24

**Authors:** Céline Elie, Magali Perret, Hayat Hage, Erwin Sentausa, Amy Hesketh, Karen Louis, Asmaà Fritah-Lafont, Philippe Leissner, Carole Vachon, Hervé Rostaing, Frédéric Reynier, Gaspard Gervasi, Adrien Saliou

**Affiliations:** 1 BIOASTER, Microbiology Research Institute, 40 avenue Tony Garnier, 69007 Lyon, France; 2 bioMérieux, 376 Chemin de l’Orme, 69280 Marcy-l’Étoile, France; 3 bioMérieux, 5 Rue des Berges, 38000 Grenoble, France

**Keywords:** Computational biology and bioinformatics, Microbiology, Molecular biology

## Abstract

The gut microbiome is widely analyzed using high-throughput sequencing, such as 16S rRNA gene amplicon sequencing and shotgun metagenomic sequencing (SMS). DNA extraction is known to have a large impact on the metagenomic analyses. The aim of this study was to compare DNA extraction protocols for 16S sequencing. In that context, four commonly used DNA extraction methods were compared for the analysis of the gut microbiota. Commercial versions were evaluated against modified protocols using a stool preprocessing device (SPD, bioMérieux) upstream DNA extraction. Stool samples from nine healthy volunteers and nine patients with a *Clostridium difficile* infection were extracted with all protocols and 16S sequenced. Protocols were ranked using wet- and dry-lab criteria, including quality controls of the extracted genomic DNA, *alpha-diversity, accuracy using a mock* community of known composition and repeatability across technical replicates. SPD improved overall efficiency of three of the four tested protocols compared with their commercial version, in terms of DNA extraction yield, sample *alpha*-diversity, and recovery of Gram-positive bacteria. The best overall performance was obtained for the S-DQ protocol, SPD combined with the DNeasy PowerLyser PowerSoil protocol from QIAGEN. Based on this evaluation, we strongly believe that the use of such stool preprocessing device improves both the standardization and the quality of the DNA extraction in the human gut microbiome studies.

## Introduction

In recent years, advances in next-generation sequencing (NGS) have revolutionized the analysis of complex microbial ecosystems including the gut microbiota, leading to advanced understanding of its role in health and disease^1–6^. Alterations in the composition and diversity of the gut microbiota communities have been correlated with a large number of diseases, such as inflammatory bowel disease^7–11^, irritable bowel syndrome^12,13^, metabolic disorders [e.g. type 2 diabetes (T2D), obesity and nonalcoholic fatty liver disease (NALFD)]^14–19^, and more recently, cancer^20–30^.

Nevertheless, metagenomics methods are known to be prone to errors at different steps of the workflow, from sample collection^31–34^, DNA extraction^35–37^, library preparation and sequencing^37–39^ to data analysis^40,41^. In order to facilitate the implementation of these methods into clinical routine practice, standardized methods are urgently needed^42–46^. Although a standard operating procedure for fecal samples DNA extraction has been provided by the International Human Microbiome Standards (IHMS, http://www.microbiome-standards.org/), the area of nucleic acids preparation is rapidly evolving and regular protocol benchmarking is required.

The choice of the DNA extraction method has been demonstrated to strongly affect the detection of bacterial communities^35,36,47–49^. DNA extraction is a sophisticated process, including sample weighing, sample homogenization, bacterial cell lysis, and DNA purification, for which each step still requires improvements and guidelines. For instance, the standard weighing procedure can be tedious and time-consuming to collect the same volume of fecal material for all samples. Sample homogenization could also have an impact on the bacteria that can be detected^50,51^. Surprisingly, few studies have reported the use of commercial devices to standardize the handling of fecal samples prior to DNA extraction^31,52,53^. Also, as the cell wall of Gram-positive bacteria is composed of a thick layer of peptidoglycan, bead-beating is now recommended to improve the lysis^37,54,55^. Nevertheless, beads can vary in size and material (e.g. ceramic, glass, zirconia or silica), which may also play a role in the lysis efficiency. Even if commercial solutions provide standardized methods for bacterial lysis and DNA purification, some laboratories still use in-house protocols, making difficult the selection of one gold-standard protocol.

Recently, twenty-one DNA extraction protocols were compared in a multicentric study across three continents using Shotgun Metagenomic Sequencing (SMS)^35^. From the analysis of two healthy individuals, the authors proposed a QIAGEN protocol, named Q, as the standardized reference protocol for DNA extraction in human gut microbiome studies. However, it has been shown that different fecal samples may vary in terms of bacterial composition (Gram-positive vs Gram-negative cells)^35,56^, microbial load (high vs low bacterial cells per fecal material)^57,58^, disease-related clinical status (healthy vs sick individuals)^59–61^ and stool consistency (separate hard lumps vs watery)^62–64^. A comparison study with a higher number of individuals including both healthy and sick donors would be of both clinical and technological interests to address the variability and heterogeneity of fecal samples.

Although SMS has the potential to deeply investigate microbial communities^65,66^, amplicon sequencing targeting the 16S rRNA gene is often the preferred and the most cost-effective metagenomic method in the analysis of clinical cohorts^67,68^. Obviously, these sequencing methods have their own limitations and biases, which are important to consider for the selection of one DNA extraction protocol in human gut microbiome studies.

To address these considerations, our study evaluated four commercially available DNA extraction methods, using 16S rRNA amplicon sequencing. These protocols were tested as recommended by the manufacturers, but also with an upstream stool preprocessing device (SPD), designed to facilitate DNA extraction^52^. The protocols were evaluated according to wet-lab as well as dry-lab criteria, using nine healthy individuals and nine *Clostridium difficile* infected (CDI) patients.

## Results

### Study design

In our study, four commercial DNA extraction protocols were evaluated based on the supplier’s recommendations: the NucleoSpin Soil kit (Macherey–Nagel, named MN), the DNeasy PowerLyzer PowerSoil kit (QIAGEN, named DQ), the QIAamp Fast DNA Stool kit (QIAGEN, named QQ), and the ZymoBIOMICS DNA Mini kit (ZymoResearch, named Z). In order to facilitate the first steps of DNA extraction, they were also tested with an upstream stool preprocessing device, named SPD (see Supplementary Methods for detailed protocols). The resulting protocols were named as follows: S-MN stands for SPD + MN, S-DQ for SPD + DQ, S-QQ for SPD + QQ and S-Z for SPD + Z.

**Figure 1:**
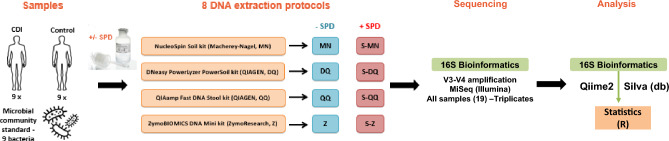
Workflow of sample processing.

We analyzed fecal samples from nine healthy volunteers (CDI−) and nine patients suffering from CDI (CDI+). A defined mixture of bacterial species (mock community) was also prepared and sequenced to assess the efficiency and accuracy of DNA extraction by comparing the observed bacterial abundances to the theoretical ones. DNA extraction protocols were compared using 16S rRNA gene amplicon sequencing for a total of 456 samples (18 fecal samples and 1 mock community, in triplicates) (Fig. 1).

### Quality and quantity of extracted DNA

When selecting a DNA extraction protocol, sufficient genomic DNA of high quality is desirable for preparing metagenomic libraries. In the present study, we evaluated the DNA yield, DNA fragment size and DNA quality. A protocol that performs poorly on these criteria would likely skew measured bacterial compositions as only a small portion of bacterial communities present in the original sample would be analyzed. A summary of DNA extraction performance for all human fecal samples is presented in Supplementary Table 1.

**Figure 2:**
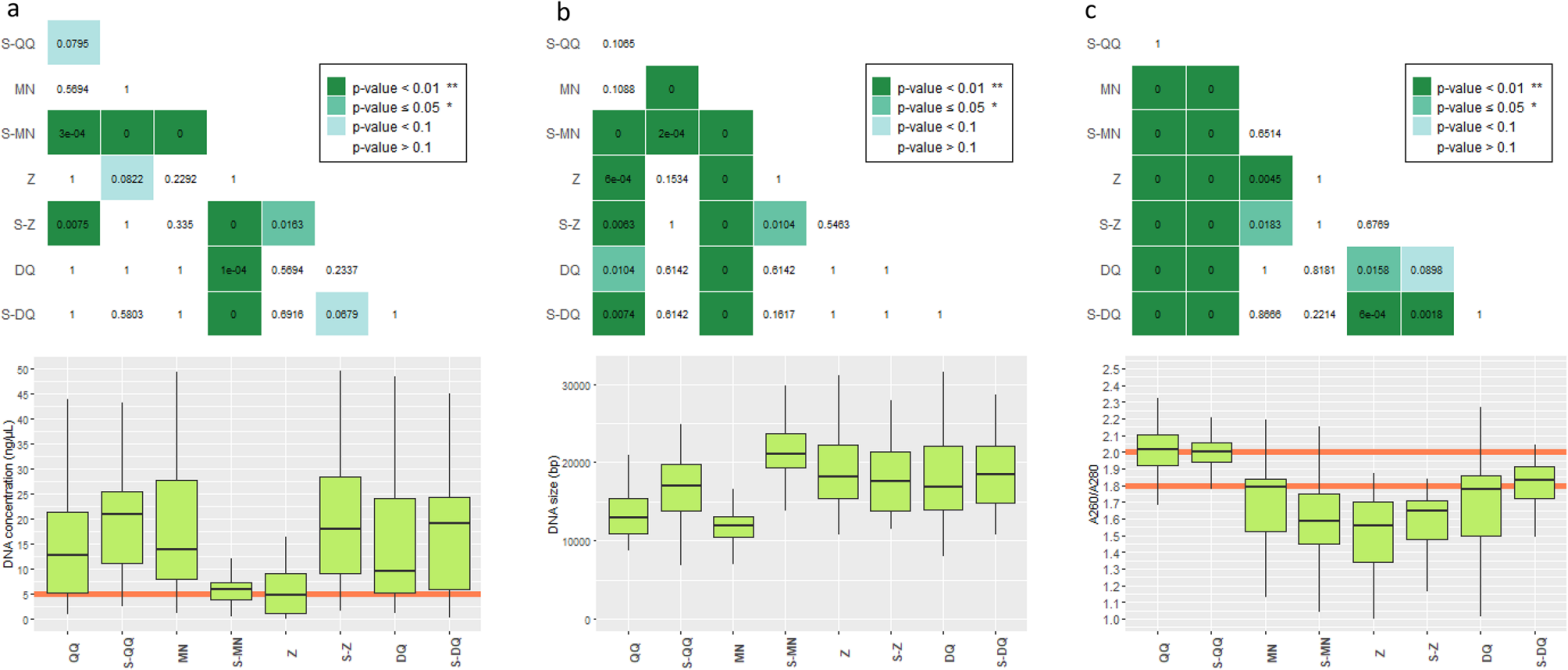
Quantity and quality of extracted DNA from human fecal samples and mock samples with eight different extraction protocols. (**a**) DNA concentration (ng/µl). The orange horizontal line represents the threshold at 5 ng/µl. (**b**) DNA size (bp). (**c**) Absorbance ratios at 260/280. DNA of good quality should have a ratio between the two orange horizontal lines (between 1.8 and 2, preferably close to 1.8). All boxplots are topped by a heatmap showing the pairwise Wilcoxon test *p*-values. Significant differences between protocols are highlighted in green.

Considerable variability was found in the extraction yield for the tested protocols (Fig. 2a), which is in line with previous studies^36^, and was not dependent on health status (Supplementary Fig. 1a). Except for MN, DNA extraction protocols in combination with SPD seemed to recover as much or more DNA compared to their commercial versions. Notably, increases were observed for S-QQ (*p*-value < 0.1) and S-Z (*p*-value < 0.05), compared to QQ and Z respectively. A same DNA yield was obtained for the protocol DQ with and without the use of SPD (*p*-value > 1). SPD seemed to negatively affect the extraction yield when coupled with the protocol MN (*p*-value < 0.01). Out of the eight extraction protocols tested, protocols S-MN and Z significantly recovered the lowest DNA concentrations.

In practice, a best performing protocol would be a protocol for which the highest number of samples could be prepared for sequencing. Here, for a given protocol, we measured the percentage of samples whose DNA concentration was superior to 5 ng/µl, threshold corresponding to the minimal DNA concentration recommended to prepare 16S rRNA gene sequencing libraries (Table 1). In our hands, none of the tested protocols was able to retrieve, for all the samples, DNA with a concentration superior to this threshold. Except for S-MN, the best performances were observed when the protocols were combined with SPD. S-Z recovered enough DNA material for 88% of samples, followed by MN (86%), S-QQ (82%) and S-DQ (81%).

**Table 1:** Performance of DNA extraction protocols regarding the percentage of human and mock samples having the required DNA input for metagenomic studies (> 5 ng/µl).

Extraction protocols	Percentage of samples > 5 ng/µl (%)
S-Z	88
MN	86
S-QQ	82
S-DQ	81
DQ	77
QQ	75
Z	56
S-MN	54

Regarding the fragment size of DNA, variations were also observed between the extraction protocols. QQ and MN protocols yielded the shortest DNA fragments with a median size around 12,000 bp, which was shorter than S-QQ (*p*-value > 0.1) and significantly shorter than the other ones (*p*-value < 0.01, Fig. 2b). The longest DNA fragment sizes were observed for S-MN, with an average size of 21,000 bp, followed by DQ, S-DQ and Z with DNA fragments around 18,000 bp (*p*-value > 0.1). DNA fragments were significantly higher in CDI positive patients when extracted with MN (*p*-value ≤ 0.05), S-QQ or DQ (*p*-value < 0.01), but other protocols showed similar DNA fragment size regardless of health status (Supplementary Fig. 1b).

We also assessed DNA purity using the A260/280 ratio. A ratio of 1.8, which is generally accepted as “pure” for DNA, was observed for S-DQ (Fig. 2c). A ratio below 1.8 was observed for the protocols MN, S-MN, Z, S-Z and DQ, which may indicate the presence of protein, phenol or other contaminants. A ratio close to 2 was assessed for QQ and S-QQ suggesting the possible presence of RNA in samples (*p*-value < 0.01 in comparison with the other protocols). Except for MN, the protocols combined with SPD generated DNA of purity equal or superior to their standard versions. Besides QQ and Z, all protocols showed equivalent DNA purity between CDI+ and CDI− samples (Supplementary Fig. 1c).

### Observed microbial diversity and performance in extracting Gram-positive bacteria

**Figure 3:**
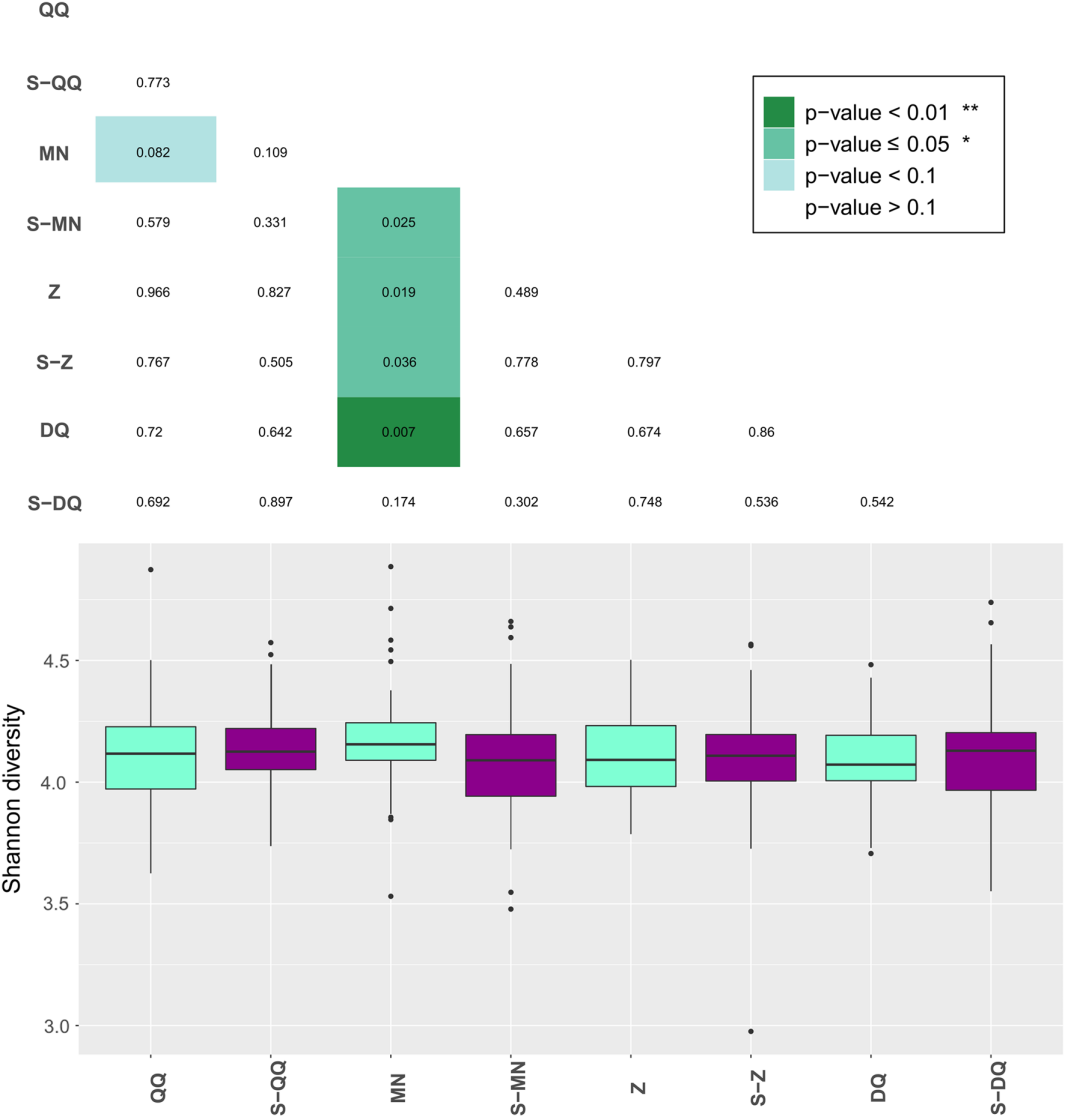
Shannon α-diversity index of human fecal sample composition. Shannon diversity calculated from the abundance table of 16S rRNA gene profiling. Standard protocols are colored in sky blue, whereas protocols associated with SPD are colored in purple. Boxplots are topped by a heatmap showing the pairwise Wilcoxon test *p*-values. Significant differences between protocols are highlighted in green.

In addition to the wet-lab criteria, the extraction quality was also evaluated, using 16S rRNA gene amplicon, by investigating the observed microbial diversity of samples (Fig. 3). This *alpha*-diversity has been recently described as a good indicator of DNA extraction performance, being positively correlated with the Gram-positive bacteria extraction^35^.

No significant difference in microbial diversity was observed for CDI+ patients compared to healthy volunteers (Supplementary Fig. 2). Previous studies have shown a significant decrease in microbial diversity in patients with recurrent CDI but not with initial CDI^69,70^. As a considerable variability was found within each group of individuals, we corrected the individual effect in the statistical model to emphasize differences between extraction protocols. The median *alpha*-diversity values were between 4.0 and 4.2 for all tested protocols (Fig. 3). Interestingly, the *alpha*-diversity was equal or the highest when samples were extracted with an SPD-associated protocol except for MN which performed better than S-MN according to 16S data (*p*-value ≤ 0.05) (Fig. 3). Preliminary SMS data also showed improved *alpha*-diversity with SPD-associated protocols compared to commercial protocols (Supplementary Fig. 3a).

We then evaluated if the observed diversity was associated with an effective Gram-positive bacteria recovery. For this purpose, we assessed the ratio *Firmicutes/Bacteroidetes*, two main phyla commonly found in the gut microbiota. *Firmicutes* and *Bacteroidetes* are phyla of bacteria, which are, for the most part, Gram-positive and Gram-negative respectively. In theory, the ratio *Firmicutes/Bacteroidetes* should be improved by a protocol performing well for the extraction of Gram-positive bacteria^71^. Remarkably, this ratio was increased for the four protocols combined with SPD in comparison to their standard versions, in both 16S and SMS data (Table 2 and Supplementary Table 2). To quantify more precisely the SPD effect on microbial community composition, DESeq2 was used to test the differential abundance of taxa between standard vs SPD-combined protocols. For each patient, the relative abundance of the *Firmicutes* phylum increased significantly, whereas the *Bacteroidetes* phylum decreased significantly with the use of SPD. This analysis was also performed at the family level (Supplementary Fig. 4), where SPD led to a significant decrease of Gram-negative families and a significant increase of Gram-positive families (Supplementary Table 4). Altogether, our results were consistent with a positive effect of SPD on the observed *alpha*-diversity by improving the recovery of Gram-positive bacteria.

**Table 2:** Estimation of the firmicutes/bacteroidetes ratio. This ratio was calculated for every individual extracted by the eight different extraction protocols using the 16S rRNA gene sequencing data (mean of triplicates).

Patient	QQ	S-QQ	MN	S-MN	Z	S-Z	DQ	S-DQ
p1 (−)	1.46	2.07	1.53	2.05	1.66	2.21	2.51	2.46
p3 (−)	0.90	2.22	0.75	1.98	0.88	2.68	1.78	2.42
p4 (−)	0.87	6.50	0.95	7.99	0.71	5.33	2.02	4.60
p5 (−)	1.56	0.91	0.88	0.79	0.92	0.87	0.77	0.85
p8 (−)	1.13	3.86	1.69	3.22	1.27	3.64	2.86	3.46
p9 (−)	1.02	5.66	1.39	8.28	1.34	11.52	2.63	4.81
p13 (−)	1.40	5.51	2.49	6.02	1.31	6.17	3.76	4.49
p14 (−)	5.19	36.32	9.67	39.66	12.25	34.76	16.88	32.88
p15 (−)	1.97	4.85	3.20	4.52	1.45	6.27	2.97	4.44
p2 (+)	2.84	25.21	3.63	21.75	2.22	20.41	7.99	18.04
p6 (+)	0.93	7.27	1.70	5.77	1.86	6.29	3.27	5.03
p7 (+)	3.98	18.99	4.92	18.68	4.03	13.13	4.84	24.97
p10 (+)	1.31	9.30	2.28	9.46	1.24	7.96	4.34	8.28
p11 (+)	0.95	1.16	0.58	1.14	0.36	1.30	0.91	0.82
p16 (+)	60.41	87.47	75.74	88.62	66.63	86.71	42.77	85.35
p17 (+)	0.75	0.92	0.58	0.90	0.49	0.94	0.64	0.94
p18 (+)	17.23	42.43	25.08	36.50	11.83	41.30	13.52	37.33
p19 (+)	41.30	61.86	43.71	64.08	30.53	56.20	28.74	63.84

### Extraction protocol accuracy

**Figure 4:**
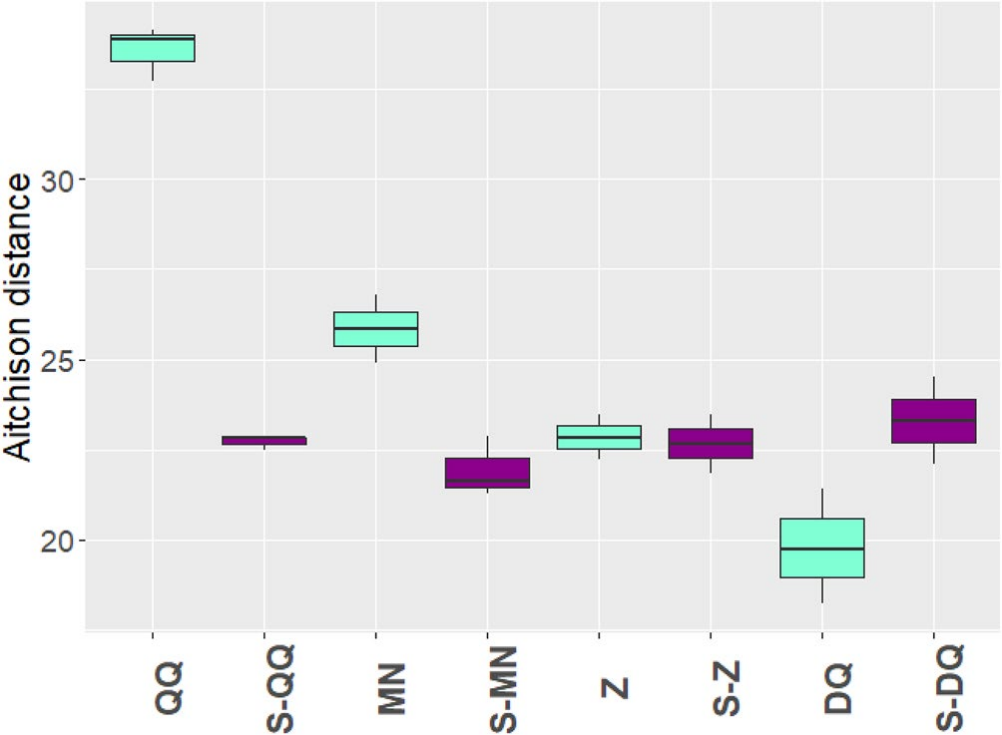
Accuracy of the observed bacterial abundance in the mock community. Aitchison distances were calculated between observed and expected bacterial abundances (CLR transformed data) in mock samples for each of three replicates of each extraction protocol, for 16S rRNA gene sequencing data.

**Figure 5:**
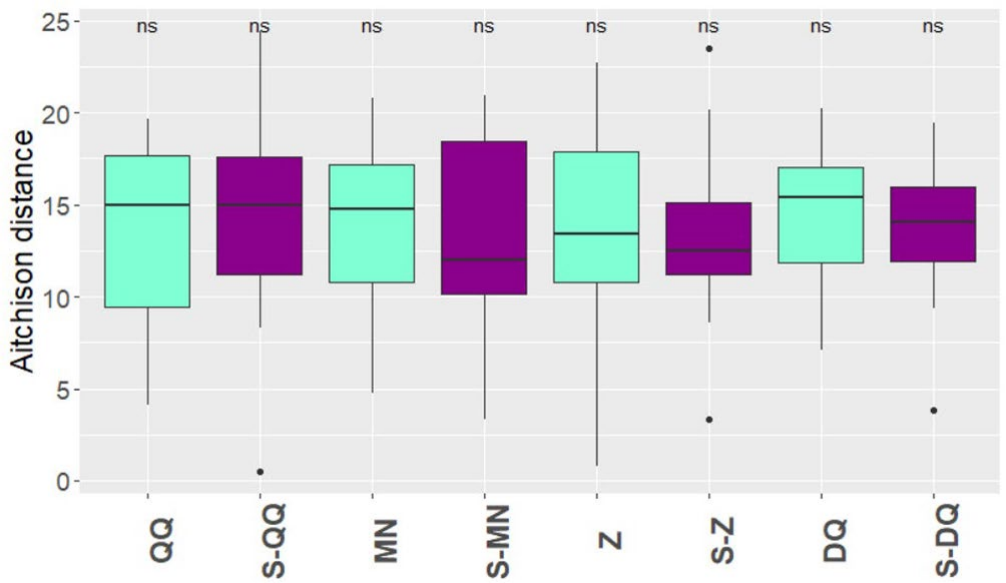
Protocol repeatability. An Aitchison distance was calculated between replicates for each genus within each human fecal sample. Standard protocols are colored in sky blue, whereas protocols with SPD are colored in purple.

In order to estimate the accuracy of the extraction protocols, a mock community consisting of nine bacterial species of known respective abundances was prepared and sequenced. The protocol accuracy was estimated by calculating the Aitchison distance (the lower the distance, the better the prediction) between observed and expected abundances at the genus level (Fig. 5). Interestingly, the bacterial abundances were better predicted using 16S rather than SMS (Fig. 4 and Supplementary Fig. 3b). Independently of the metagenomics methods, these predictions were improved when SPD was used upstream for the protocols QQ and MN. Based on 16S rRNA gene data, DQ was the most accurate protocol, followed by S-MN, S-Z, Z and S-QQ. Detailed bacterial abundances at the genus level are plotted in Supplementary Fig. 4. As observed for human samples, SPD improved the recovery of Gram positive bacteria compared to standard protocols. Discrepancies between expected and observed abundances seem mostly related to GC content^72^. Considering both approaches, bacterial families with high GC content such as *Pseudomonas* tend to be overestimated whereas families with low GC content such as *Listeria* tend to be underestimated. However, this pattern is not as visible with SPD-associated protocols.

### Protocol repeatability

The eight protocols were next evaluated for repeatability across the variations of bacterial abundances between triplicates of a same stool sample (Fig. 5). We observed an increase of the repeatability when the protocols were coupled with SPD compared to their standard versions except for QQ but this increase was not significant (*p*-value > 0.1). The median of the Aitchison distance was divided by 1.01 between QQ (14.99) and S-QQ (14.90), 1.08 between Z (13.44) and S-Z (12.40), 1.22 between MN (14.70) and S-MN (12.01) and 1.09 between DQ (15.30) and S-DQ (14.05). S-MN was the most repeatable protocol, closely followed by S-Z.

### Protocols overall performance

**Figure 6:**
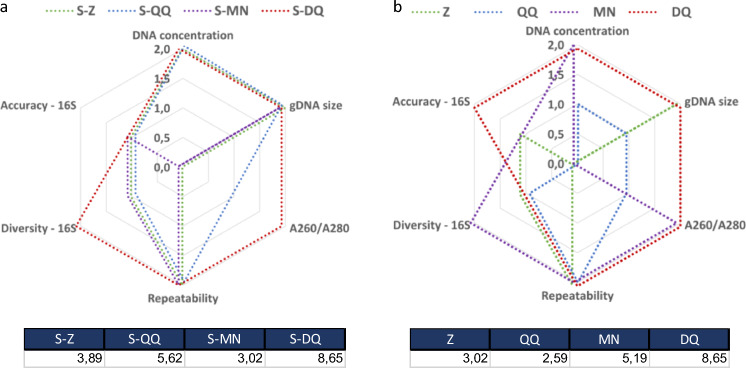
Overall performance of the eight extraction protocols based on studied criteria using 16S rRNA gene profiling. (**a**) Protocols in combination with SPD. (**b**) Standard protocols (no SPD). A score of 0, 1 or 2 was given to each protocol. Scores of 0 correspond to the weakest values, whereas scores of 2 are the best values of the considered criteria. The area value obtained for each protocol is indicated in the tables below each chart.

In our study, eight DNA extraction protocols were evaluated using both wet- and dry-lab criteria, with 16S rRNA sequencing read-outs. To help in data interpretation, we ranked the protocols according to a custom designed scoring system which was assigned to each criterion based on the observed 16S rRNA gene profiling results (Fig. 6). For each criterion, a score of 0 (the worst result obtained in our dataset), 1 or 2 (the best result obtained in our dataset) was given. These scores were then plotted using a spider chart: a score of 0 represents the center, whereas a score of 2 is the vertex. Protocols were given the same score if no significant difference was observed. The generated areas were then used to help in selecting the best-overall performing DNA extraction protocol.

The protocols Z and QQ combined with SPD performed better compared to their standard version while performance of the MN protocol was diminished when combined with the SPD (Fig. 6). Protocols S-DQ and DQ showed equivalent overall performances for the represented criteria with S-DQ showing higher microbial diversity and DQ, improved accuracy. Considering SPD associated protocols, in our hands, S-DQ showed the best overall performance (Fig. 6a). Although other protocols showed similarly good results for some criteria, S-DQ was the only protocol being among the best performing protocols for all tested criteria. The S-DQ performance was slightly inferior to S-QQ and S-Z regarding DNA yield but this difference was not significant (*p*-value > 0.05, Fig. 2a). Even if S-DQ was not the best protocol for this criterion, enough DNA material was produced for more than 80% of samples to prepare and sequence the metagenomics libraries. S-DQ was also found to be less repeatable than S-Z and S-MN but the slight differences were not significant (Fig. 5).

Considering the standard versions of the protocols, DQ had the best overall performance (Fig. 6b). This protocol performed well in terms of accuracy and extracted DNA yield and quality. MN performed significantly better than DQ for microbial diversity (*p*-value < 0.01, Fig. 3), but performed poorly on other criteria. Finally, MN, QQ and Z were slightly more repeatable than DQ, but not significantly (*p*-value > 0.1, Fig. 6).

## Discussion

DNA extraction is a crucial step of the metagenomics workflow known to be influenced by many parameters, which are difficult to evaluate exhaustively. In addition to in-house protocols, new commercial solutions are now emerging, making difficult the choice of a good protocol for the gut microbiota. Benchmarking protocols is thus crucial to understand the potential biases and to avoid errors during data interpretation. Recent gut microbiome studies compared various DNA extraction protocols but were limited to a low number of fecal samples, mainly from healthy individuals^27–30,37,73,74^. As a consequence, the performance of such protocols may not be guaranteed for a clinical cohort.

Our study is the first, to our knowledge, to compare four commercial DNA extraction protocols using 16S rRNA amplicon sequencing method on an adequate number of stool samples for statistical analysis and biological conclusion (n = 18). In an effort to streamline fecal preparation prior to DNA extraction, the commercial protocols were also tested in combination with a stool preprocessing device. As recommended by recent studies, we also included a positive control, the mock community, so that we could reliably assess the accuracy of extraction protocols. The mock was made up of nine bacterial species and processed alongside fecal specimens. The eight protocols tested were ranked based on wet- and dry-lab criteria. The global aim was to identify one method that performs well and generates the most accurate and reproducible data.

In addition to healthy donors, patients suffering from a *Clostridium difficile* Infection were also recruited, allowing to test the protocols on samples with various microbial composition, consistency and biomass. CDI is a burning issue, as *Clostridium difficile*, a Gram-positive bacterium, is the leading cause for diseases from mild diarrhea to pseudomembranous colitis in hospitalized patients^75^. Fecal microbiota transplant (FMT) is emerging as a new option for recurrent CDI^76^. Identifying which bacteria are already present (recipient) and have been transferred (donor) is essential and requires the use of highly sensitive, robust and fast metagenomics techniques^70,77^.

In our study, a total of 456 and 56 samples were analyzed using 16S rRNA gene sequencing and SMS respectively, allowing to have an important dataset for comparison results. Even if, as expected, SMS is more sensitive in bacterial detection, our present findings indicate good agreement between the two sequencing methods. However, it is to be noted that only one replicate of the SMS experiment was performed and further validation is needed. Our data also show good agreement between the samples from the two groups of individuals. Interestingly, our results show that no single DNA extraction protocol performed best on all the criteria tested. However, differences were not all significant, and considering the strategy of selection described above, the standard DQ protocol and S-DQ appeared as the best-performing protocols among commercial and SPD-associated solutions for extracting DNA from human fecal samples. The DQ protocol with or without the SPD generated an amount of good quality DNA that was compatible with subsequent library preparations for all samples. Extracted DNA quantity was superior to 5 ng/μl for 81% and 77% of samples using S-DQ and DQ respectively. Regarding the dry-lab criteria, for 16S rRNA profiling, DQ showed improved accuracy whereas S-DQ combined the best results in terms of *alpha-diversity, extraction of Gram-positive bacteria*, repeatability and accuracy in bacterial detection.

Remarkably, the bioinformatics analysis also shed light on the added value of the stool preprocessing device for some extraction protocols. In our study, the protocols in combination with SPD have in common the first steps of the procedure. This includes the shaking and the mechanical lysis with zirconia and silica beads 0.1 mm. In such combination, we observe an increase of the observed *alpha*-diversity. Our results are in good agreement with Costea et al. who showed that these parameters of the protocol were positively associated with the observed diversity, which is a good indicator of an efficient lysis^35^. Biased protocols are also known to cause overrepresentation of Gram-negative bacteria due to the inefficient lysis of Gram-positive bacteria. For the SPD-combined protocols, we observed an increase of the relative abundance of Gram-positive bacteria and a corresponding decrease in the relative abundance of Gram-negative bacteria, which led to an increase of the *Firmicutes/Bacteroidetes* ratio. The SPD can therefore provide more accurate characterization of the microbiota by reducing the ratio bias. In terms of repeatability, SPD also showed promising results. This device would be of particular interest to limit variations when several experimenters, and even different labs in case of multi-centric studies, perform DNA extraction. Other approaches such as the OMNIgene^®^•GUT system (DNA Genotek) or RNA*later* (Thermo Fisher) preservation tubes also exhibit higher DNA extraction yield compared to snap-frozen samples (Neuberger-Castillo et al*.*, 2020), further highlighting the added value of sample preprocessing. Lastly, the use of our in-house mock community, composed of both Gram-positive and Gram-negative bacteria cells, made possible to benchmark the protocols in terms of bacterial abundance predictions. Our results demonstrate that SPD in combination with most of the tested protocols is more accurate in assessing the bacterial abundances than the protocols in their standard versions. Comparison of the performance of the SPD device used in this study with other sample preprocessing methods is required to establish a new standard method. Such device prior DNA extraction may add additional costs and extra time and labor to the DNA extraction reactions but, from our perspective, getting unbiased and comparable microbiome data across labs and countries is priceless.

In this study, we focused on sample preprocessing and commercial solutions for DNA extraction. However, several other steps such as sample homogenization and library preparation are also crucial for accurate microbial community profiling. We are also aware that all the protocols may not have been tested in optimal parameters. The commercial protocols were tested using the beads provided in the kit on a Retsch system for 5 min. In our hands, protocol Z was one of the worst performer according to wet-lab criteria. Today, Zymo Research recommends other bead-beating protocols than the one tested. As shown by Tourlousse et al*.*, vigorous bead-beating regimes allows effective recovery of Gram-positive bacteria. Optimizing this step may, therefore, improve extraction performance of all methods^37,78^. In a similar way the DNeasy PowerSoil kit (Catalog No. 12888-100), a previous version of the DNeasy PowerLyzer PowerSoil kit (Catalog No. 12855-100), was compared to other commercial solutions including the NucleoSpin Soil kit by Yang et al*.* In their hands, the QIAGEN protocol showed a lesser performance than the other protocols unlike the most recent kit which performed best in our study. This highlights the difficulty to establish a gold-standard for gut microbiome analysis with the numerous, ever-evolving protocols. Moreover, great progress is been made in the field of automated nucleic acid extraction. Assessing performance of such systems would also be relevant in the scope of clinical studies.

## Conclusion

We recommend the S-DQ protocol to extract microbial DNA from human stool samples. While we have only tested S-DQ on fecal samples, we suppose that it might also work well with other types of microbiota samples, although some modifications may be necessary.

In addition to the DNA extraction protocol, sample preprocessing appears to be a new way to improve the overall performance of most DNA extraction protocols. We propose to now include stool-preprocessing devices in new microbiome studies to streamline and standardize DNA extraction.

## Methods

### Ethics approval and consent to participate

Fecal samples used in this study corresponds to left-over samples collected for diagnostic purpose. Each patient was informed regarding collection, storage and use for research activities. As this study was out of the regulations related to clinical trials, non-opposition statement was obtained from all subjects and was sufficient to process the fecal samples according to the French legal and medical ethical guidelines. Both collection and use of fecal samples for metagenomic analyses were authorized by the French Ministry of Higher Education, Research and Innovation (Declaration N°DC-2018-3240).

### Stool samples

Fecal samples from nine healthy volunteers and nine patients with *Clostridium difficile* infection (CDI) were provided by a certified testing laboratory in France and tested for *Clostridium difficile* toxins. Upon reception, each fecal sample was freshly aliquoted into 24 tubes (8 protocols × 3 replicates) and frozen at − 80 °C until extraction, the − 80 °C storage being known to maintain a stable microbial community for long-term period^79^.

### Microbial mock community

The microbial mock community was prepared by mixing nine bacteria (Table 3), including four easy-to-lyse Gram-negative bacteria (*Pseudomonas aeruginosa*, *Escherichia coli*, *Salmonella enterica and Rhizobium radiobacter*) and five more difficult to lyse Gram-positive bacteria (*Lactobacillus fermentum*, *Enterococcus faecalis*, *Staphylococcus aureus*, *Listeria inocula* and *Bacillus subtilis*). Bacterial cells were obtained from ATCC and cultivated according to ATCC’s recommendations. The number of viable cells was estimated by plate counting. The mock community was prepared by mixing between 2.7 × 10^7^ and 3.6 × 10^8^ cells of nine bacteria and stored at − 80 °C until extraction.

**Table 3:** Composition of the microbial mock community and culture conditions.

Bacterial strain	ATCC number	Gram strain	Medium (Agar/Broth)	Temperature (°C)	Number of cells
*Pseudomonas aeruginosa*	ATCC^®^ 9027-MINI-PACK™	−	Nutrient	37	1.00E+08
*Escherichia coli*	ATCC^®^ 8739-MINI-PACK™	−	Nutrient	37	1.38E+08
*Agrobacterium radiobacter*	ATCC^®^ 53487™	−	Nutrient	30	3.50E+07
*Salmonella enterica*	ATCC^®^ 700147™	−	Trypticase Soy	37	3.60E+08
*Lactobacillus fermentum*	ATCC^®^ 11739™	+	Lactobacilli MRS	37	7.70E+07
*Enterococcus faecalis*	ATCC^®^ 29212-MINI-PACK™	+	Brain heart infusion	37	1.50E+08
*Staphylococcus aureus*	ATCC^®^ BAA-977-MINI-PACK™	+	Brain heart infusion	37	5.80E+07
*Listeria innocua*	ATCC^®^ 33090™	+	Brain heart infusion	37	1.10E+08
*Bacillus subtilis*	ATCC^®^ 19659-MINI-PACK™	+	Nutrient	30	2.70E+07

### DNA extraction

Four commercial protocols were compared in this study, according to the manufacturers’ recommendations: the NucleoSpin Soil kit (#740780.50, protocol May 2016/Rev. 06, Macherey–Nagel), the DNeasy PowerLyzer PowerSoil Kit (#12855-100, protocol 07272016, QIAGEN), the QIAamp Fast DNA Stool kit (#51604, QIAGEN, protocol modified from Ref.^36^) and the ZymoBIOMICS DNA Mini kit (#D4300, protocol 1.1.0, ZymoResearch). These protocols were also tested in combination with a stool preprocessing device (SPD, #421061, bioMérieux^52^). This device was designed to facilitate and standardize fecal sample preparation before nucleic acid extraction. It includes a spoon for a 200 mg calibrated sample and a vial containing a buffer for sample resuspension, glass beads for homogenization and two filters for retaining fecal debris. After 5 min hands-on-time, the filtrate is ready-to-use for downstream DNA extraction. Protocols of extraction methods as well as SPD are detailed in Supplementary Methods. DNA was extracted in triplicates from fecal samples and from the microbial community. A260/A280 ratio was assessed using the DropSense 96 system (Trinean). Genomic DNA size was assessed using the Genomic DNA ScreenTape (#5067-5364, Agilent) on the 2200 TapeStation system (Agilent). DNA concentrations were estimated using the QuantiFluor One dsDNA kit (#E4870, Promega) with the GloMax system (Promega).

### 16S rRNA gene library preparation and sequencing

16S rRNA gene libraries were prepared according to Illumina’s protocol (# 15044223 RevB^80^). In order to minimize the risk of cross-contamination and pipetting errors, the workflow was automated using a high-throughput liquid handler; the Freedom EVO NGS workstation (TECAN)^81^. Briefly, V3-V4 hypervariable regions were first amplified from 12.5 ng of genomic DNA, using the following primers: (i) Forward Primer: TCGTCGGCAGCGTCAGATGTGTATAAGAGACAGCCTACGGGAGGCAGC-AG and (ii) Reverse Primer: GTCTCGTGGGCTCGGAGATGTGTATAAGAGACAGGACTACHVG-GGTWTCTAAT and 2X KAPA HiFi HotStart ReadyMix (Kapa Biosystems). PCR cycle conditions were 95 °C for 3 min, 25 cycles of (95 °C for 30 s, 55 °C for 30 s 72 °C for 30 s), then a final extension of 72 °C for 5 min. The libraries were purified using AMPure XP beads (Beckman Coulter). Dual indexes and sequencing adapters from the Illumina Nextera XT index kits (Illumina) were added in a second PCR using 2× KAPA HiFi HotStart ReadyMix (Kapa Biosystems). Cycle conditions were 95 °C for 3 min, 8 cycles of (95 °C for 30 s, 55 °C for 30 s, 72 °C for 30 s), then a final extension of 72 °C for 5 min. Ready-to-sequence libraries were purified using AMPure XP beads (Beckman) and quantified by fluorescence using the QuantiFluor One dsDNA kit (# E4870, Promega) with the GloMax system (Promega). Quality control was performed using a 2200 TapeStation system with the DNA 1000 ScreenTape (# 5067-5582, Agilent). The library pool was quantified by qPCR with the KAPA Library Quantification Kit for Illumina platforms (Kapa Biosystems). Sequencing was performed on a MiSeq system (Illumina) with the MiSeq Reagent v3 kit (600 cycles) in a 2 × 300 bp mode.

### Shotgun metagenomic library preparation and sequencing

SMS libraries were prepared using the Nextera XT DNA Library Preparation Kit (# FC-131-1096, Illumina), following Illumina’s instructions (protocol # 15031942 v03 February 2018). Briefly, 1 ng of genomic DNA was used for the tagmentation reaction for a total volume of 20 µl. After 5 min at 55 °C, the reaction was stopped by adding 5 µl of the Neutralize Tagment (NT) Buffer. A limited-cycle PCR amplification was then performed to amplify the tagmentated DNA [addition of 15 μl of Nextera PCR Master Mix (NPM)] and to add Illumina sequencing adapters (addition of 5 µl of both Index 1 primer and Index 2 primer from the Nextera XT index kit, Illumina) for a total volume of 50 µl. The following PCR cycle program was used: 72 °C for 3 min, 95 °C for 30 s, 12 cycles of (95 °C for 10 s, 55 °C for 30 s, 72 °C for 30 s), 72 °C for 5 min. SMS libraries were quantified using the QuantiFluor One dsDNA kit (# E4870, Promega) with the GloMax system (Promega). The quality of libraries was assessed using the High Sensitivity DNA kit on the Agilent 2100 Bioanalyzer. Sequencing was performed on a NextSeq500 system (Illumina) with the NextSeq 500/550 High Output v2 kit (300 cycles) in 2 × 150 bp.

### 16S rRNA gene profiling

The analysis was done using Snaq^82^, a snakemake pipeline for 16S data analysis with QIIME2. Briefly, quality trimming was done using bbduk (BBTools) with a quality threshold of 20. During the PCR amplification process, artefactual sequences can be generated from multiple parent sequences, and are called chimeric sequences. These sequences were removed using the DADA2^83^ algorithm, which, in addition, joins paired end reads and produce Amplicon Sequence Variant tables (ASVs). The taxonomy assignment of ASVs was done using the “feature-classifier” plugin with SILVA classifier trained on V3 and V4 regions (cls-silvaV34^84,85^).

### Shotgun metagenomic profiling

After quality control with FastQC (v0.11.9), reads were trimmed and filtered based on the sequence quality and length using fastp (v0.20.0) with the default parameters. Contamination with host DNA was discarded by mapping the filtered reads on the human reference genome version GRCh37 using BBMap (v38.90)^86^. Clean reads were annotated using the kraken2 software (v2.1.1)^87^ against the Unified Human Gastrointestinal Genome (UHGG) catalog^88^.

### Statistical analysis

All analyses were performed in R (version 3.3.1). The analysis of microbiome compositional data were done on centered log-ratio (CLR) transformed matrices using the clr function from the “compositions” R package. The repeatability was assessed by calculating a Aitchison distance between replicates of a condition for every patient. *Alpha*-diversity (Shannon indices) was calculated for each sample using the vegan package. The taxonomical analysis of the mock community samples was done by mapping their SMS and 16S data using bowtie2 (v.2.3.5.1)^89^ on indexes created with the 9 expected species (Table 3). The accuracy of the protocols was evaluated on those samples by calculating the Euclidean distance between expected and predicted abundances after CLR transformation using the “philentropy” R package. Differentially abundant bacteria between protocols with or without the SPD were identified using the DESeq2 package. For each criterion (except for *alpha*-diversity), the statistical significance of the differences between protocols was computed with a pairwise Wilcoxon rank test. For multiple comparisons, *p-*values were corrected by Benjamini Yakuteli correction and adjusted *p-*values below 0.05 were considered statistically significant. The *alpha*-diversity values varied greatly from one patient to another, so the patient effect was controlled in a linear model using the “limma” package, and statistics were computed with the empirical Bayes method.

## Supplementary Information

Supplementary Information 1.

Supplementary Information 2.

## Data Availability

The datasets generated during the current study are available on the BioProject database (ID PRJNA648321), at the following link: http://www.ncbi.nlm.nih.gov/bioproject/648321.

## Abbreviations

SMS: Shotgun metagenomic sequencing
SPD: Stool preprocessing device
T2D: Type 2 diabetes
NAFLD: Nonalcoholic fatty liver disease
Q: Protocol QIAGEN from ^35^
CDI: *Clostridium difficile Infection*
PCR: Polymerase chain reaction
NPM: Nextera PCR master mix
OTU: Operational taxonomic units
NT: Neutralize tagment
MN: The NucleoSpin Soil kit (Macherey–Nagel)
DQ: The DNeasy PowerLyzer PowerSoil kit (QIAGEN)
QQ: The QIAamp Fast DNA Stool kit (QIAGEN)
Z: The ZymoBIOMICS DNA Mini kit (ZymoResearch)
S-MN: SPD in combination with MN
S-DQ: SPD in combination with DQ
S-QQ: SPD in combination with QQ
S-Z: SPD in combination with Z
