# Supplementary material for: Comparison of DNA extraction methods for 16S rRNA gene sequencing in the analysis of the human gut microbiome
Source: Sci Rep. 2023 Jun 24;13:10279. doi: 10.1038/s41598-023-33959-6 (PMC10290636; doi:10.1038/s41598-023-33959-6)
Supplement: Supplementary file 1 — Supplementary Information 1. [file 41598_2023_33959_MOESM1_ESM.docx]

**Comparison of DNA extraction methods for 16S rRNA gene a sequencing in the analysis of the human gut microbiome**

Céline Elie^1^, Magali Perret^1^, Hayat Hage^1^, Erwin Sentausa^1^, Amy Hesketh^1^, Karen Louis^1^, Asmaà Fritah-Lafont^1^, Philippe Leissner^1^, Carole Vachon^3^, Hervé Rostaing^3^, Frédéric Reynier^1^, Gaspard Gervasi^2^, and Adrien Saliou^1^*

***Corresponding author**

**Affiliations**

^1^BIOASTER, Microbiology Research Institute, 40 avenue Tony Garnier, 69007 Lyon, France

^2^bioMérieux, 376 Chemin de l'Orme, 69280 Marcy-l'Étoile, France

^3^bioMérieux, 5 Rue des Berges, 38000 Grenoble, France

**Running title**: Comparison of DNA extraction methods for the human gut microbiome

**Supplementary Methods**

**DNA extraction protocol # DQ**

PowerLyzer PowerSoil DNA Isolation Kit (Mobio)

DNeasy PowerLyzer PowerSoil Kit (Qiagen, Catalog number 12855-100)

1. To the PowerLyzer Glass Bead Tube, 0.1 mm provided, add 200 mg feces

2. Add 750 µl of Bead Solution to the Glass Bead Tube. Gently vortex to mix

3. Incubate the samples 10 min at 65 °C, then 10 min at 95 °C

4. If Solution C1 is precipitated, heat to 60 °C until dissolved before use

5. Add 60 μl of Solution C1 and vortex briefly

6. Bead beating: Transfer the samples in Retsch 5 min at 30 Hz

7. Transfer the supernatant to a clean 2 ml Collection Tube. Expect ~400-500 μl supernatant

8. Add 250 μl of Solution C2 and vortex for 5 sec. Incubate at 4 °C for 5 min

9. Spin the tubes at room temperature for 1 minute at 10,000 x g

10. Transfer no more than, 600 μl of supernatant to a 2 ml Collection Tube

11. Add 200 μl of Solution C3 and vortex briefly. Incubate at 4 °C for 5 minutes

12. Spin tubes at 10,000 x g for 1 minute at room temperature

13. Transfer no more than 750 μl of supernatant to a 2 ml Collection Tube

14. Add 1200 μl of Solution C4 to the supernatant and vortex for 5 seconds

15. Load 675 μl onto a Spin Filter and spin at 10,000 x g for 1 minute at room temperature

16. Discard the flow through. Repeat a total of three times to process all sample

17. Add 500 μl of Solution C5 and spin for 30 seconds at 10,000 x g

18. Discard the flow through

19. Centrifuge again at room temperature for 1 minute at 10,000 x g

20. Place Spin Filter in a clean 1.5 ml tube avoiding splashing Solution C5 onto the Spin Filter

21. Add 100 μl of sterile DNA-Free PCR Grade Water to the center of the white filter membrane

22. Spin for 30 seconds at 10,000 x g

23. Discard the filter and store DNA at -20 °C or -80 °C

**DNA extraction protocol # QQ**

DNA Isolation QIAamp Fast DNA Stool (Qiagen) modified. Knudsen et al., mSystems 2016

1. Weigh 0.2 g of feces into a bead beating tube, and place the tube on ice

2. Add 1 ml InhibitEX Buffer to the tube containing the sample aliquot

3. Vortex continuously until the sample is thoroughly homogenized

4. Treat sample in a Retsch at 30 Hz for 5 min

5. Heat the sample at 95 °C for 7 min

6. Vortex the sample for 15 s

7. Centrifuge the sample at full speed for 1 min to pellet sample particles

8. Pipet 30 μl proteinase K into a new 1.5 ml microcentrifuge tube (not provided)

9. Pipet 400 μl supernatant from step 7 into the 1.5 ml microcentrifuge tube containing

proteinase K

10. Add 400 μl Buffer AL and vortex for 15 s

11. Incubate at 70 °C for 10 min. Centrifuge briefly to remove drops from the inside of the tube lid

12. Add 400 μl of ethanol (96–100%) to the lysate, and mix by vortexing. Centrifuge briefly to remove drops from the inside of the tube lid

13. Carefully apply 600 μl lysate from step 12 to the QIAamp spin column. Close the cap and centrifuge at full speed for 1 min

14. Apply the remaining lysate from step 12 to the spin column, close the cap and centrifuge at full speed for 1 min. Place the spin column in a new 2 ml collection tube, and discard the tube containing the filtrate

15. Carefully open the QIAamp spin column and add 500 μl Buffer AW1. Centrifuge at full speed for 1 min. Place the QIAamp spin column in a new 2 ml collection tube, and discard the collection tube containing the filtrate

16. Carefully open the QIAamp spin column and add 500 μl Buffer AW2. Centrifuge at full speed for 3 min. Discard the collection tube containing the filtrate

17. Place the QIAamp spin column in a new 2 ml collection tube (not provided) and discard the old collection tube with the filtrate. Centrifuge at full speed for 3 min

18. Transfer the QIAamp spin column into a new, labeled 1.5 ml low-DNA bind microcentrifuge tube (not provided) and pipet 100 μl Buffer ATE directly onto the QIAamp membrane. Incubate for 3 min. at room temperature, then centrifuge at full speed for 1 min to elute DNA

19. Store DNA at -20 °C or -80 °C until further use

**DNA extraction protocol # Z**

ZymoBIOMICS™ DNA Miniprep Kit (Zymo Research)

1. Add sample to a ZR BashingBead™ Lysis Tubes (0.1 & 0.5 mm). Add 750 μl ZymoBIOMICS™ Lysis Solution to the tube and cap tightly

2. Treat sample in a Retsch at 30 Hz for 5 min

3. Centrifuge the ZR BashingBead™ Lysis Tubes (0.1 & 0.5 mm) in a microcentrifuge at ≥ 10,000 x g for 1 minute

4. Transfer up to 400 μl supernatant to the Zymo-Spin™ III-F Filter in a Collection Tube and centrifuge at 8,000 x g for 1 minute. Discard the Zymo-Spin™ III-F Filter

5. Add 1,200 μl of ZymoBIOMICS™ DNA Binding Buffer to the filtrate in the Collection Tube from Step 4. Mix well

6. Transfer 800 μl of the mixture from Step 5 to a Zymo-Spin™ IIC-Z Column in a Collection Tube and centrifuge at 10,000 x g for 1 minute

7. Discard the flow through from the Collection Tube and repeat Step 6

8. Add 400 μl ZymoBIOMICS™ DNA Wash Buffer 1 to the Zymo-Spin™ IIC-Z Column in a new Collection Tube and centrifuge at 10,000 x g for 1 minute. Discard the flow-through

9. Add 700 μl ZymoBIOMICS™ DNA Wash Buffer 2 to the Zymo-Spin™ IIC-Z Column in a Collection Tube and centrifuge at 10,000 x g for 1 minute. Discard the flow-through

10. Add 200 μl ZymoBIOMICS™ DNA Wash Buffer 2 to the Zymo-Spin™ IIC-Z Column in a Collection Tube and centrifuge at 10,000 x g for 1 minute

11. Transfer the Zymo-Spin™ IIC-Z Column to a clean 1.5 ml microcentrifuge tube and add 100 μl ZymoBIOMICS™ DNase/RNase Free Water directly to the column matrix and incubate for 1 minute. Centrifuge at 10,000 x g for 1 minute to elute the DNA

12. Place a Zymo-Spin™ III-HRC Filter in a new Collection Tube and add 600 μl ZymoBIOMICS™ HRC Prep Solution. Centrifuge at 8,000 x g for 3 minutes

13. Transfer the eluted DNA (Step 11) to a prepared Zymo-Spin™ III-HRC Filter in a clean 1.5 ml microcentrifuge tube and centrifuge at exactly 16,000 x g for 3 minutes

14. Store DNA at -20 °C or -80 °C until further use

**DNA extraction protocol # MN**

NucleoSpin® Soil (Macherey-Nagel)

1. Transfer 250 mg fresh sample material to a NucleoSpin® Bead Tube Type A containing the ceramic beads

2. Add 700 μL Buffer SL1 or Buffer SL2

3. Add 150 μL Enhancer SX and close the cap

5. Transfer the NucleoSpin® Bead Tubes to a Retsch instrument for 5 min at 30 Hz

4. Centrifuge for 2 min at 11,000 x g

5. Add 150 μL Buffer SL3 and vortex for 5 s. Incubate for 5 min at 4 °C. Centrifuge for 1 min at 11,000 x g

6. Place a NucleoSpin® Inhibitor Removal Column (red ring) in a Collection Tube (2 mL, lid). Load up to 700 μL clear supernatant onto the filter. Centrifuge for 1 min at 11,000 x g

7. Add 250 μL Buffer SB and close the lid. Vortex for 5 s

8. Place a NucleoSpin® Soil Column (green ring) in a Collection Tube (2 mL). Load 550 μL sample onto the column. Centrifuge for 1 min at 11,000 x g. Discard flow-through and place the column back into the collection tube. Load the remaining sample onto the column. Centrifuge for 1 min at 11,000 x g. Discard flow-through and place the column back into the collection tube

9. Add 500 μL Buffer SB to the NucleoSpin® Soil Column. Centrifuge for 30 s at 11,000 x g. Discard flow-through and place the column back into the collection tube

10. Add 550 μL Buffer SW1 to the NucleoSpin® Soil Column. Centrifuge for 30 s at 11,000 x g. Discard flow-through and place the column back into the collection tube

11. Add 700 μL Buffer SW2 to the NucleoSpin® Soil Column. Close the lid and vortex for 2 s. Centrifuge for 30 s at 11,000 x g. Discard flow-through and place the column back into the collection tube

12. Add 700 μL Buffer SW2 to the NucleoSpin® Soil Column. Close the lid and vortex for 2 s. Centrifuge for 30 s at 11,000 x g. Discard flow-through and place the column back into the collection tube

13. Centrifuge for 2 min at 11,000 x g

14. Place the NucleoSpin® Soil Column into a new microcentrifuge tube. Add 100 μL Buffer SE to the column. Do not close the lid and incubate for 1 min at room temperature (18–25 °C). Close the lid and centrifuge for 30 s at 11,000 x g

15. Store DNA at -20 °C or -80 °C until further use

**Stool Preprocessing Device (SPD) protocol**

The SPD protocol is based on 4 steps:

- a sampling of the fresh stool specimen with a calibrated spoon

- a suspension and filtration of the stool to eliminate the dirty matrix

- a lysis of the bacterial cells

- and the use of one commercial extraction protocol

1. Mix and collect the sample with the spoon

2. Spoonful, remove excess of stool. Add 1 spoon of the stool sample into the SPD bottle. Screw the sample

3. Clip the devices onto the vortex Genie 2, using the vortex adaptor for homogenization (8 positions, bioMerieux). The sticker on the device cap should be oriented upwards. Vortex 2 min at maximum speed, at room temperature for stool suspending and homogenization

4. Break the “butterfly” spout of the cap and put the end rod above an Eppendorf 2 mL

5. Filtrate the liquefied matrix by several manual pressures of the soft device bottle to fill the Eppendorf tube 2 mL

6. Centrifuge the tube at room temperature at 12,000 rpm during 3 min

7. Discard the supernatant

8. Resuspend the pellet with 600 µl TE Buffer. Vortex the tube 10 sec at maximum speed for a complete resuspension

9. Transfer the 600 µl in a lysis tube containing beads

10. Clip the lysis tube onto the Mobio vortex with a microtube adaptor

11. Vortex 15 min at full speed. Wait 1 min

12. To extract DNA using the commercial kits, use the following table:

|  | DNA extraction protocols | | | |
| --- | --- | --- | --- | --- |
|  | protocol # DQ | protocol # QQ | protocol # Z | protocol # MN |
| Starting step | 13 | 8 | 4 | 6 |
| Supernatant volume | 480 µl | 400 µl | 400 µl | 480 µl |
| Solution to add to the supernatant | 768 µl solution C4 | 30 µl PK + 400 µL AL | 1,200 µl of ZymoBIOMICS™ DNA Binding Buffer + 1/200 B-mercaptoethanol | 171 µl SB |
