# Supplementary material for: Comparison of DNA extraction methods for 16S rRNA gene sequencing in the analysis of the human gut microbiome
Source: Sci Rep. 2023 Jun 24;13:10279. doi: 10.1038/s41598-023-33959-6 (PMC10290636; doi:10.1038/s41598-023-33959-6)
Supplement: Supplementary file 2 — Supplementary Information 2. [file 41598_2023_33959_MOESM2_ESM.docx]

**Supplementary Figures**

c

b

a

**
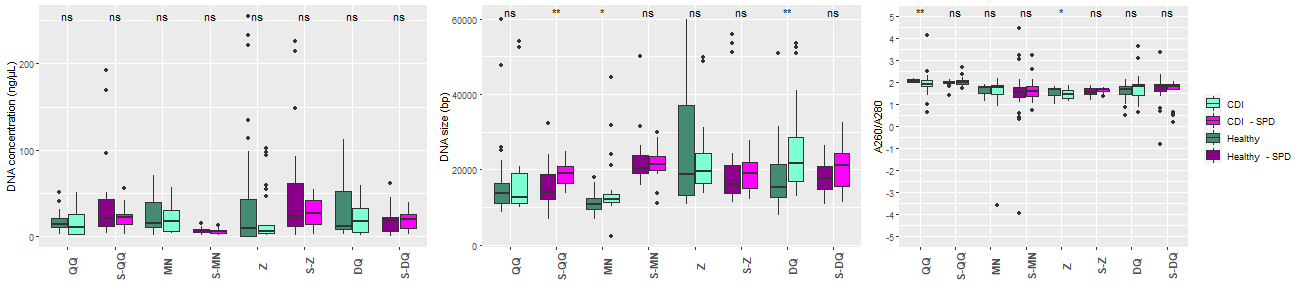
**

**Supplementary Figure 1. Quantity and quality of extracted DNA from CDI patient (dark hues) and healthy volunteer (light hues) fecal samples with eight different extraction protocols.** (a) DNA concentration (ng/µL). (b) DNA size (bp). (c) Absorbance ratios at 260/280. Ns: *p*-value > 0.05; *: *p*-value ≤ 0.05; **: *p*-value < 0.01 (pairwise Wilcoxon test).

b

a


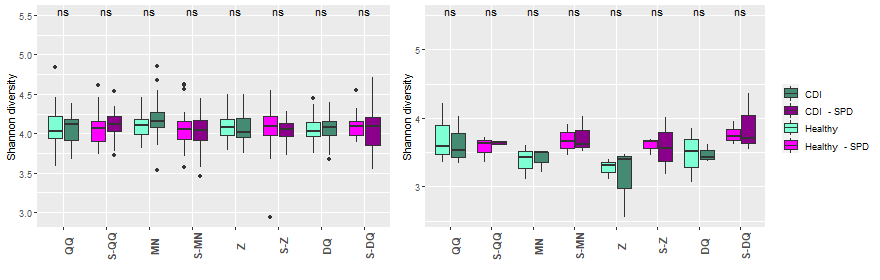


**Supplementary Figure 2. Shannon α-diversity index of human fecal sample composition from CDI patient (dark hues) and healthy volunteer (light hues) using 16S rRNA gene profiling (a) or SMS (b)** (ns. Non-significant; pairwise Wilcoxon test).

b

a


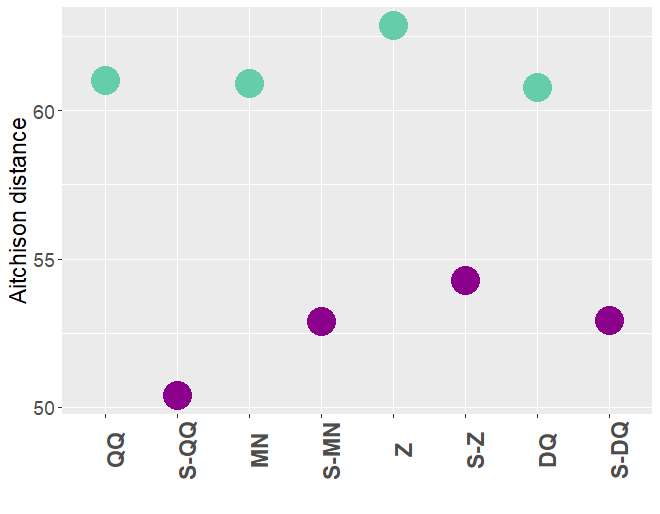

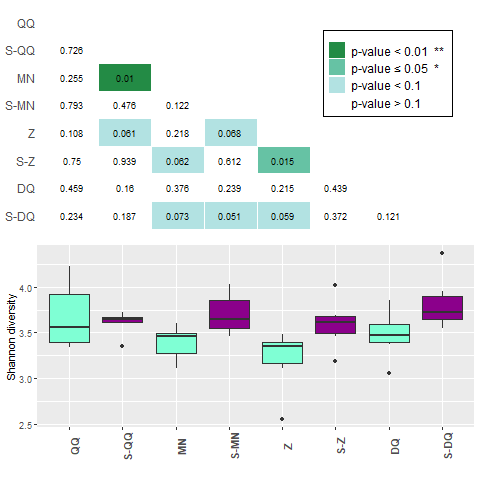


**Supplementary Figure 3. DNA extraction protocol assessment with SMS read-out for 56 samples including one replicate for 3 CDI- individuals, 3 CDI+ and 1 mock community.** Standard protocols are colored in sky blue, whereas protocols associated with SPD are colored in purple. (a) Shannon *α*-diversity index calculated from the abundance table of SMS data at species level. Boxplots are topped by a heatmap showing the pairwise Wilcoxon test *p*-values, generated in R version 4.1.0 using ggplot2 package. The statistical significant test were computed with a pairwise Wilcoxon rank test. Significant differences between protocols are highlighted in green. (b) Accuracy of the observed bacterial abundance. Aitchison distances were calculated between observed and expected bacterial abundances in mock samples.

**Supplementary Figure 4. Bacterial taxonomy at the family level from 16S data for each patient.
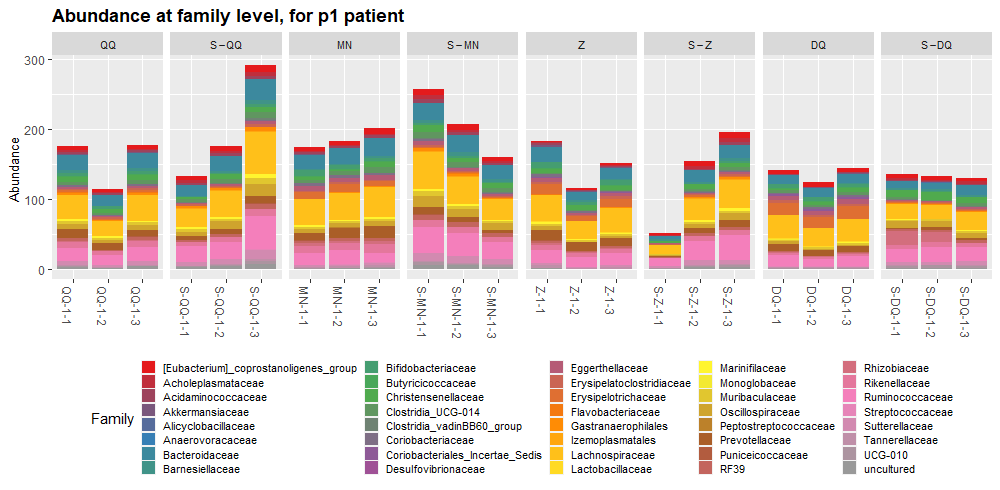
**


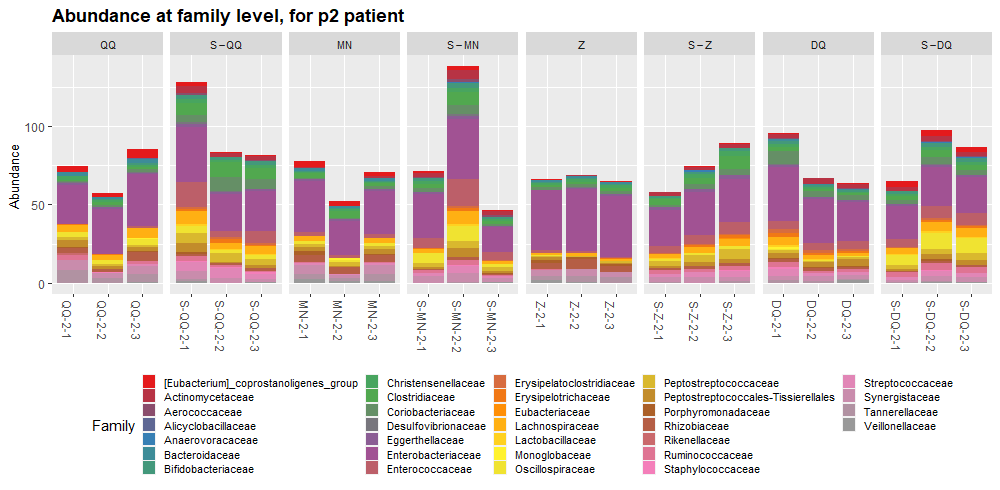

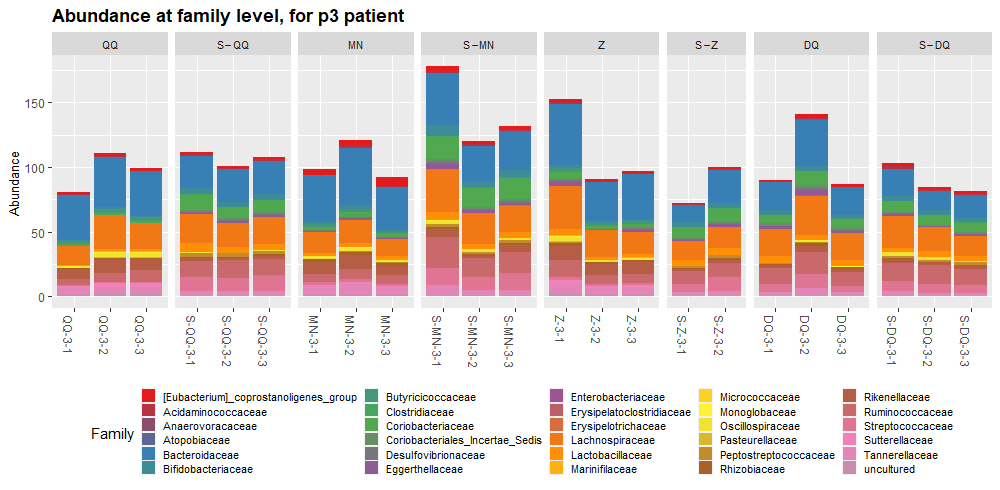

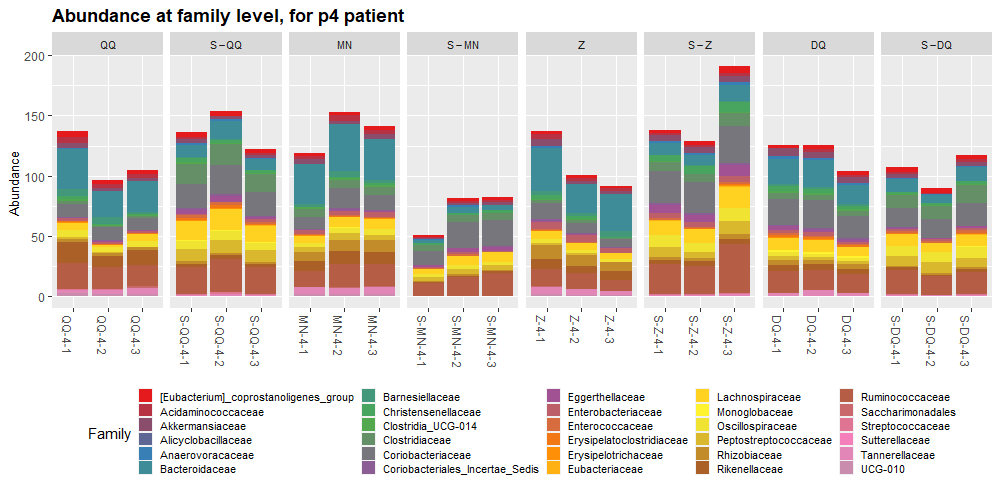

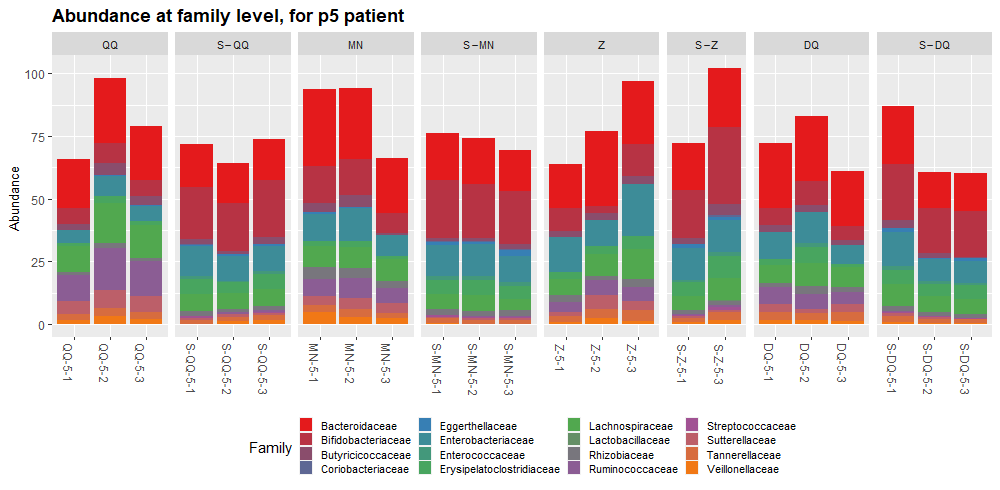

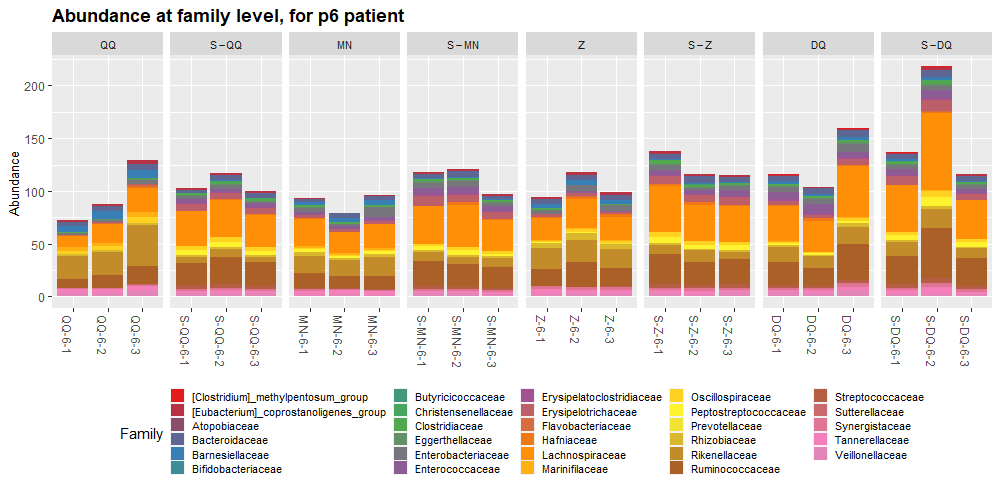

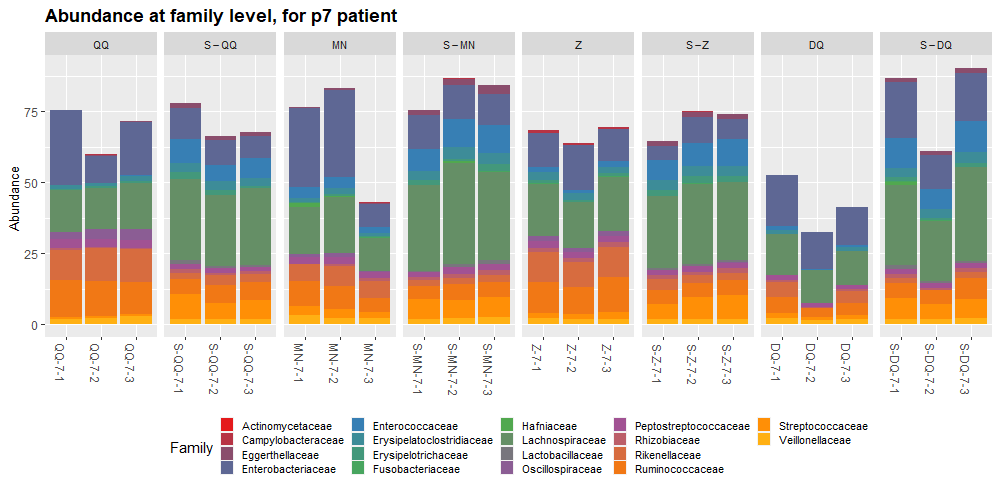

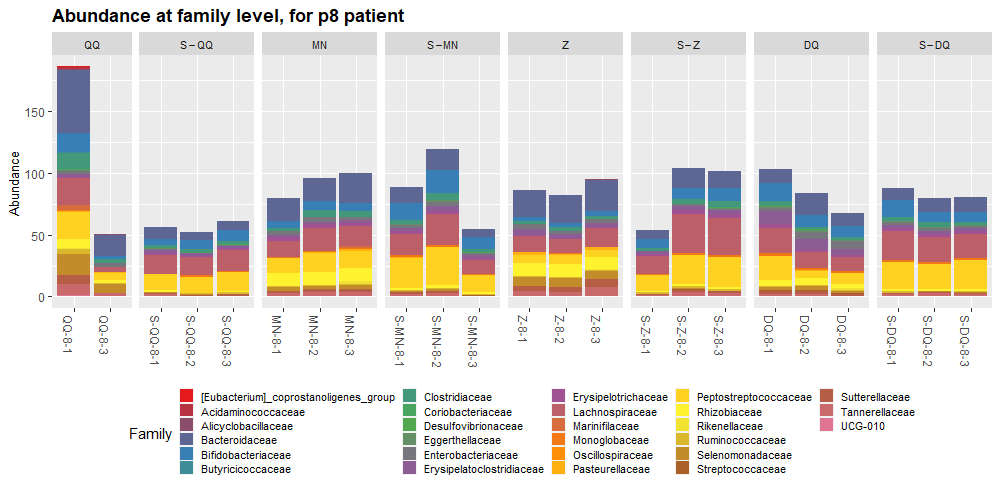

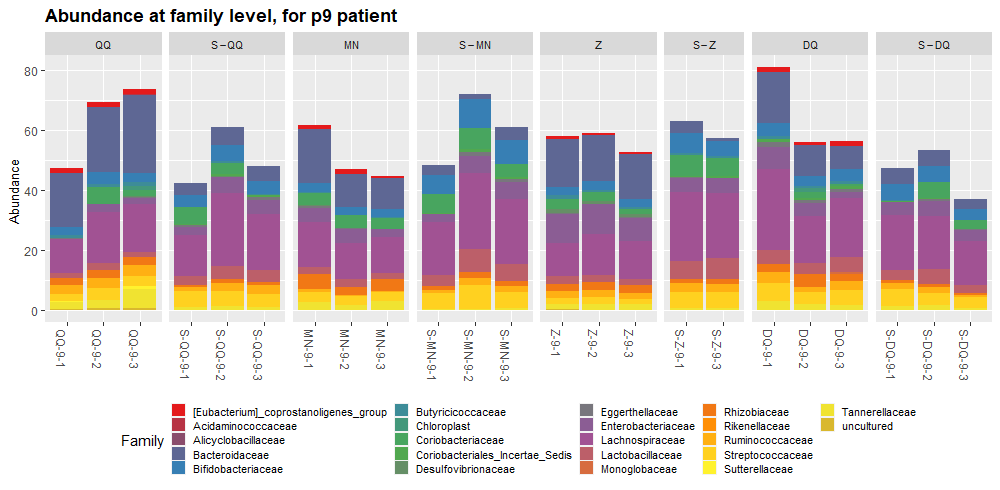

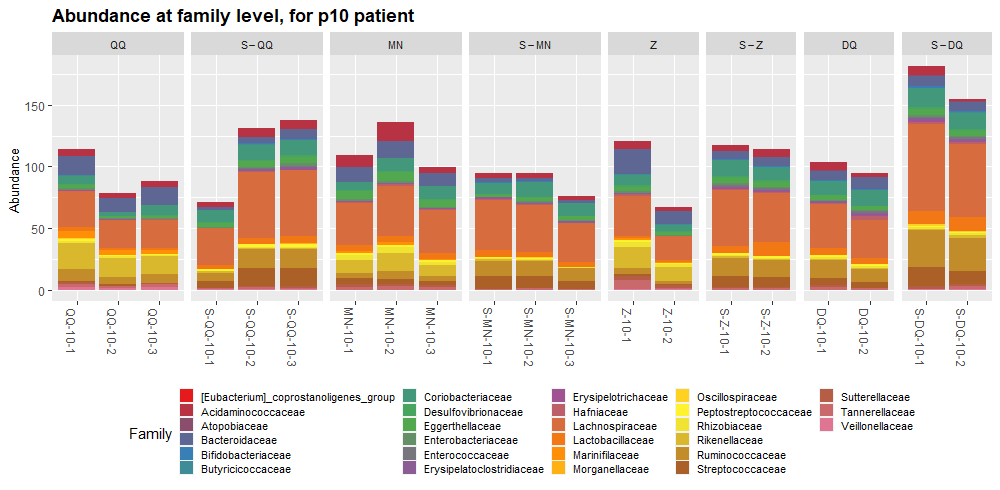

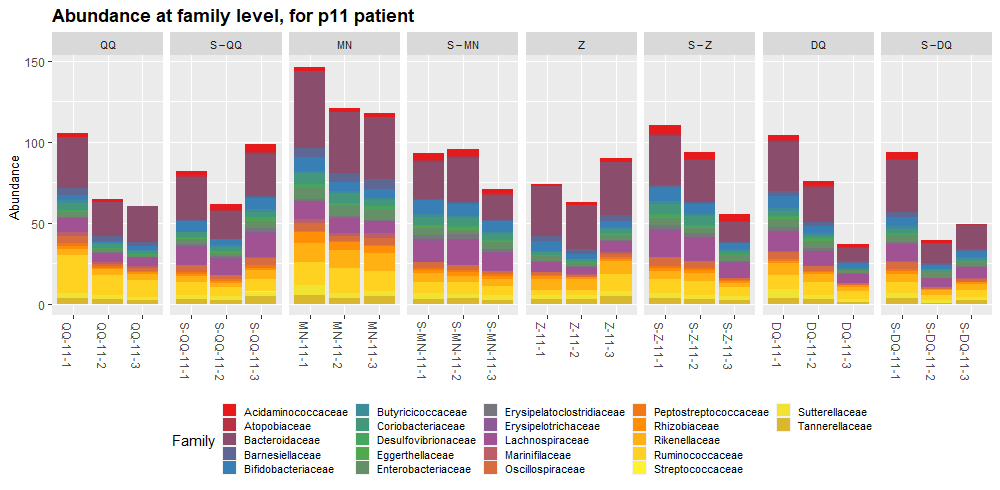

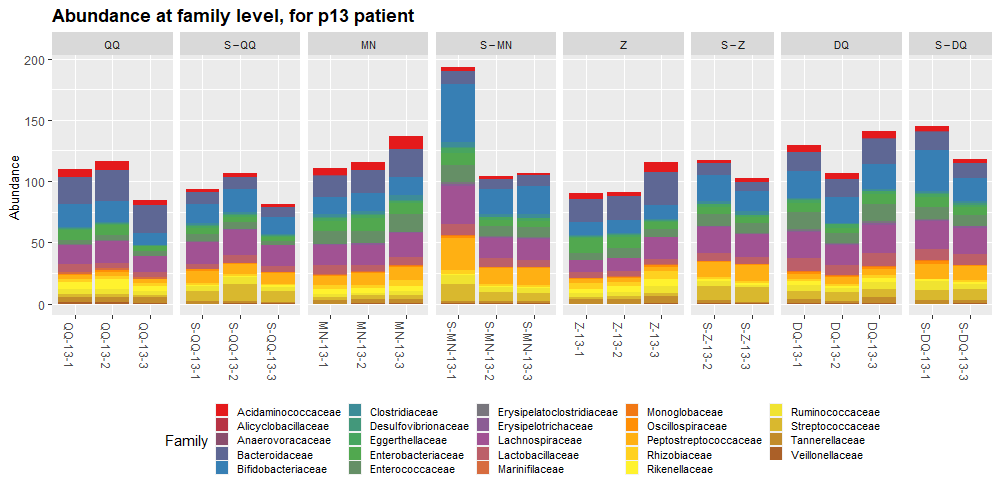

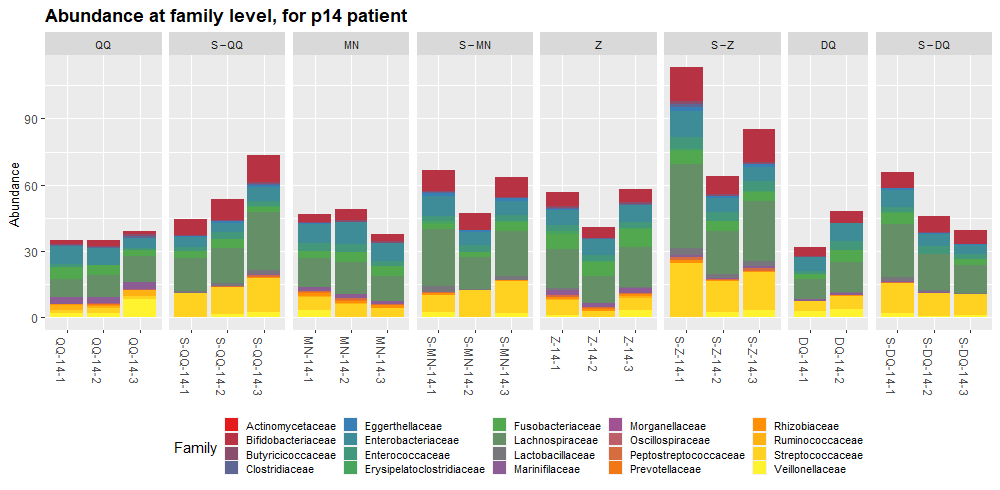

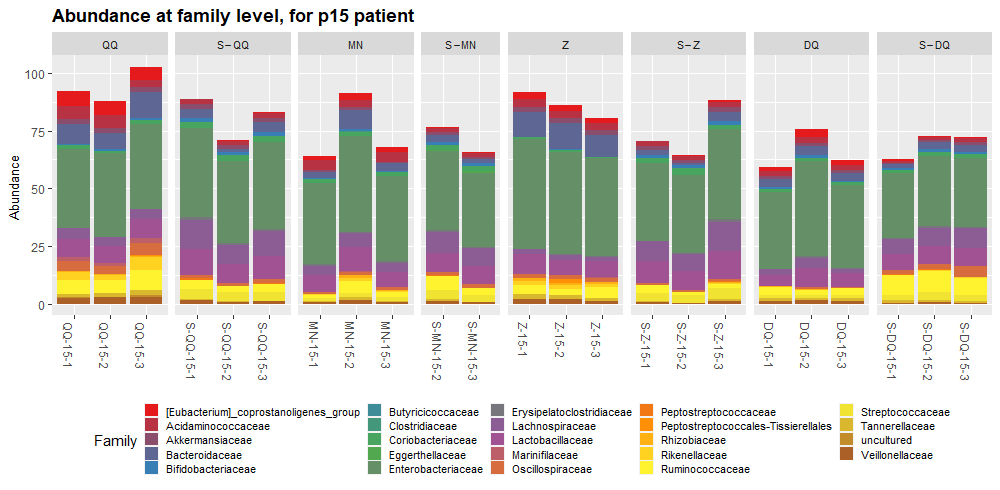

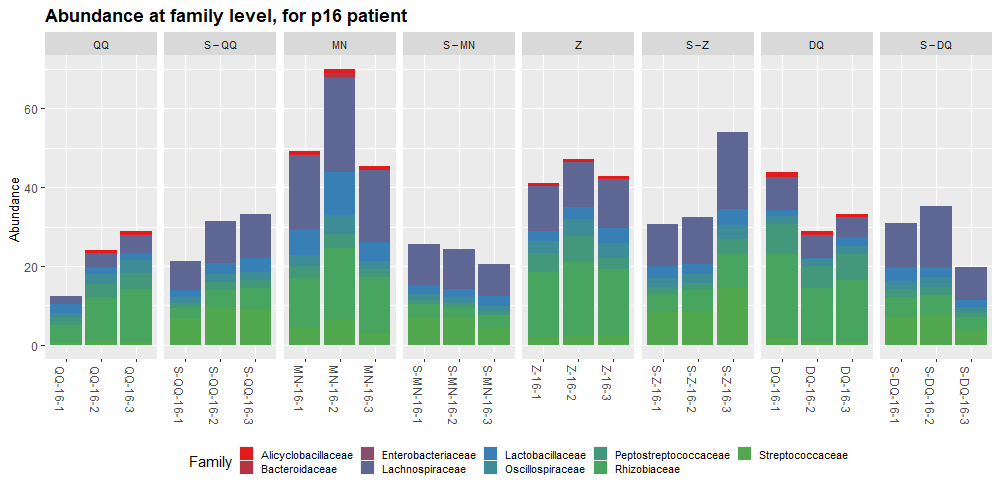

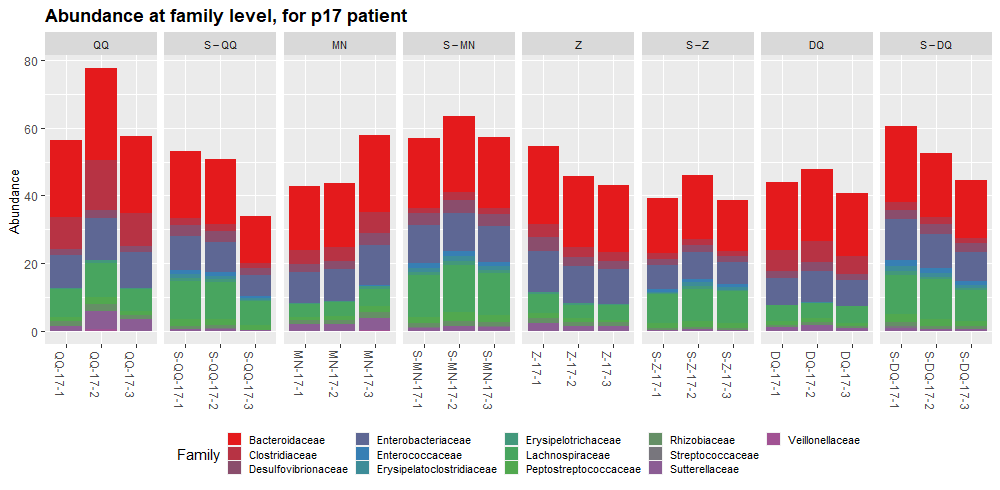

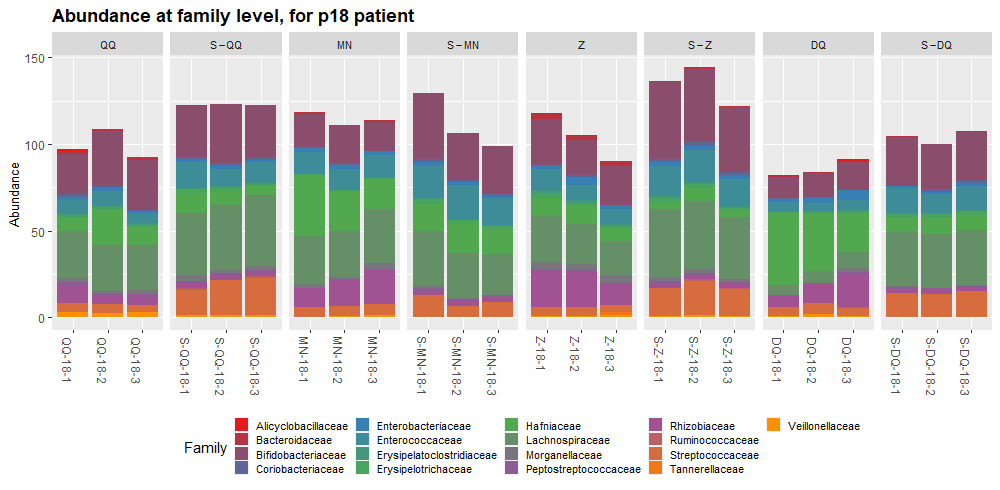

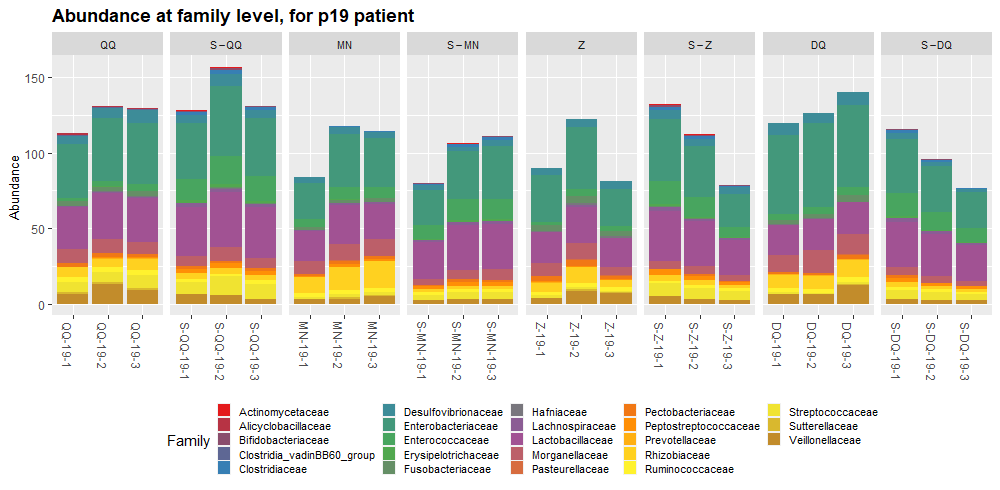


**
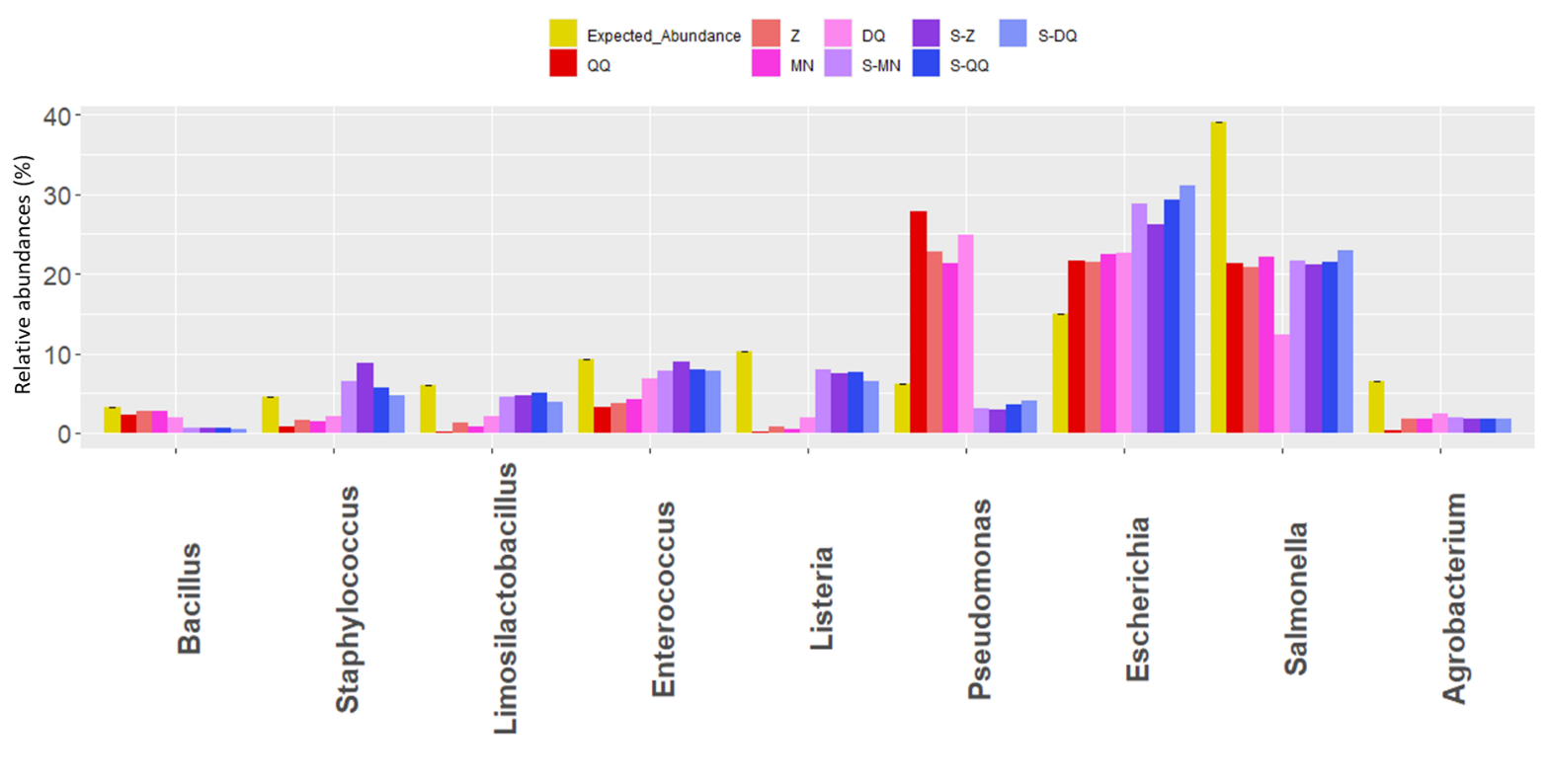

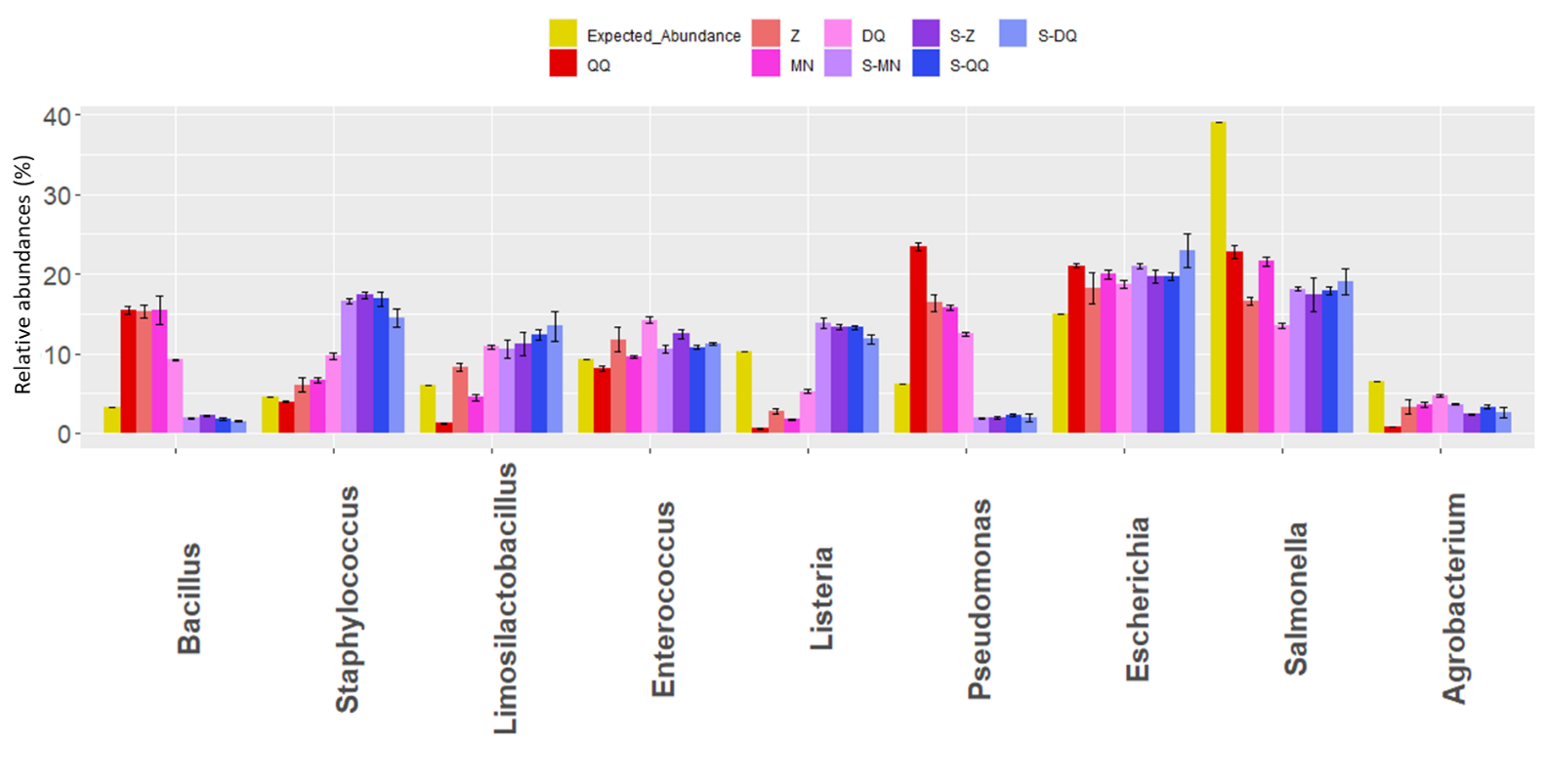
**

b

b

a

a

b

**Supplementary Figure 5. Comparison of the eight extraction protocols on their abundance prediction accuracy at genus level, for 16S (a) and SMS (b) data.** Expected relative abundances of each of the nine bacteria in the mock are represented in yellow.

**Supplementary Table**

**Supplementary Table 1. Summary table of DNA extraction performance on patient and healthy volunteer samples**

|  | **Sample ID** | **CDI status** | **Fragment Size (bp)** | **Ratio A260/A280** | **Yield (ng/µL)** |
| --- | --- | --- | --- | --- | --- |
| **Mock** | MN-mock-1 | Negative | 12911 | 1.94 | 14.95 |
|  | MN-mock-2 | Negative | 12353 | 1.9 | 11.41 |
|  | MN-mock-3 | Negative | 10989 | 1.93 | 12.89 |
|  | DQ-mock-1 | Negative | 22294 | 1.98 | 9.22 |
|  | DQ-mock-2 | Negative | 22035 | 1.94 | 9.01 |
|  | DQ-mock-3 | Negative | 24534 | 1.95 | 10.17 |
|  | QQ-mock-1 | Negative | 22824 | 2.27 | 2.68 |
|  | QQ-mock-2 | Negative | 24282 | 1.61 | 3.68 |
|  | QQ-mock-3 | Negative | 20299 | 2.23 | 3.53 |
|  | Z-mock-1 | Negative | 37735 | 1.52 | 5.77 |
|  | Z-mock-2 | Negative | 21646 | 1.59 | 6.86 |
|  | S-MN-mock-1 | Negative | 17583 | 2.17 | 3.6 |
|  | S-MN-mock-2 | Negative | 13571 | -2.55 | 4.3 |
|  | S-MN-mock-3 | Negative | 13900 | 1.46 | 4.3 |
|  | S-DQ-mock-1 | Negative | 22736 | 1.83 | 5.23 |
|  | S-DQ-mock-2 | Negative | 21043 | 1.79 | 6.11 |
|  | S-DQ-mock-3 | Negative | 19658 | 2.03 | 5.26 |
|  | S-QQ-mock -2 | Negative | NA | 1.87 | 3.8 |
|  | S-QQ-mock-1 | Negative | 15484 | 1.7 | 3.65 |
|  | S-QQ-mock-3 | Negative | 16740 | 1.94 | 4.75 |
|  | S-Z-mock-1 | Negative | 16999 | 1.38 | 2.4 |
|  | S-Z-mock-2 | Negative | NA | 1.53 | 1.79 |
|  | S-Z-mock-3 | Negative | NA | 1.4 | 1.65 |
| **Patient 1** | MN-1-1 | Negative | NA | 1.37 | 1.19 |
|  | MN-1-2 | Negative | 17880 | 1.41 | 1.63 |
|  | MN-1-3 | Negative | 16617 | 1.34 | 1.57 |
|  | DQ-1-1 | Negative | 16880 | 1.23 | 2.42 |
|  | DQ-1-2 | Negative | 21519 | 1.46 | 5.86 |
|  | DQ-1-3 | Negative | 18145 | 1.61 | 2.86 |
|  | QQ-1-1 | Negative | 17828 | 2 | 23.11 |
|  | QQ-1-2 | Negative | 15470 | 2.01 | 14.12 |
|  | QQ-1-3 | Negative | 16810 | 2.04 | 16.92 |
|  | Z-1-1 | Negative | 12062 | 1.63 | 0 |
|  | Z-1-2 | Negative | NA | 1.51 | 0 |
|  | Z-1-3 | Negative | 19198 | 1.71 | 0 |
|  | S-MN-1-1 | Negative | NA | 1.1 | 1.25 |
|  | S-MN-1-2 | Negative | NA | 1.2 | 1.43 |
|  | S-MN-1-3 | Negative | NA | 1.54 | 1.34 |
|  | S-DQ-1-1 | Negative | NA | 2.38 | 0.3 |
|  | S-DQ-1-2 | Negative | NA | 3.35 | 0.45 |
|  | S-DQ-1-3 | Negative | NA | -0.82 | 0.6 |
|  | S-QQ-1-1 | Negative | 9571 | 1.84 | 25.31 |
|  | S-QQ-1-2 | Negative | 8336 | 1.95 | 12.35 |
|  | S-QQ-1-3 | Negative | 8487 | 1.8 | 17.6 |
|  | S-Z-1-1 | Negative | 11465 | 1.68 | 60.28 |
|  | S-Z-1-2 | Negative | 13192 | 1.84 | 88.35 |
|  | S-Z-1-3 | Negative | 12568 | 1.74 | 58.36 |
| **Patient 2** | MN-2-1 | Positive | 44551 | 1.92 | 11.59 |
|  | MN-2-2 | Positive | 2374 | 1.05 | 8.01 |
|  | MN-2-3 | Positive | 31830 | 0.9 | 10.69 |
|  | DQ-2-1 | Positive | 40987 | 1.26 | 40.45 |
|  | DQ-2-2 | Positive | 50983 | 1.36 | 39.96 |
|  | DQ-2-3 | Positive | 28455 | 1.52 | 41.5 |
|  | QQ-2-1 | Positive | 12587 | 1.56 | 6.92 |
|  | QQ-2-2 | Positive | 11500 | 1.72 | 6.24 |
|  | QQ-2-3 | Positive | 11584 | 2.18 | 3.53 |
|  | Z-2-1 | Positive | 18409 | 1.59 | 13.19 |
|  | Z-2-2 | Positive | 19538 | 1.24 | 9.31 |
|  | Z-2-3 | Positive | 22150 | 1.25 | 7.49 |
|  | S-MN-2-1 | Positive | 21443 | 0.73 | 6.82 |
|  | S-MN-2-2 | Positive | 20928 | 1.2 | 3.18 |
|  | S-MN-2-3 | Positive | 21763 | 1.33 | 6.82 |
|  | S-DQ-2-1 | Positive | 14608 | 0.6 | 2.12 |
|  | S-DQ-2-2 | Positive | 11244 | 0.62 | 2.6 |
|  | S-DQ-2-3 | Positive | 12890 | 0.6 | 2.38 |
|  | S-QQ-2-1 | Positive | 13821 | 1.93 | 36.87 |
|  | S-QQ-2-2 | Positive | 16963 | 1.96 | 26.23 |
|  | S-QQ-2-3 | Positive | NA | 1.91 | 25.33 |
|  | S-Z-2-1 | Positive | 15055 | 1.72 | 8.1 |
|  | S-Z-2-2 | Positive | 14814 | 1.67 | 27.66 |
|  | S-Z-2-3 | Positive | 12515 | 1.57 | 49.63 |
| **Patient 3** | MN-3-1 | Negative | 10074 | 1.47 | 6.91 |
|  | MN-3-2 | Negative | 13781 | 1.4 | 10.08 |
|  | MN-3-3 | Negative | 13064 | 1.7 | 9.9 |
|  | DQ-3-1 | Negative | 31526 | 1.46 | 9.56 |
|  | DQ-3-2 | Negative | 50837 | 1.18 | 7.97 |
|  | DQ-3-3 | Negative | NA | 1.68 | 6.14 |
|  | QQ-3-1 | Negative | NA | 1.94 | 31.46 |
|  | QQ-3-2 | Negative | 47790 | 2.04 | 21.5 |
|  | QQ-3-3 | Negative | 60000 | 2 | 28.78 |
|  | Z-3-1 | Negative | 60000 | 1.8 | 0 |
|  | Z-3-2 | Negative | 53956 | 1.71 | 0 |
|  | Z-3-3 | Negative | 50177 | 1.75 | 0 |
|  | S-MN-3-1 | Negative | 19559 | 1.5 | 15.03 |
|  | S-MN-3-2 | Negative | 19682 | 1.44 | 10.25 |
|  | S-MN-3-3 | Negative | 21121 | 1.59 | 11.92 |
|  | S-DQ-3-1 | Negative | 21169 | 1.82 | 24.73 |
|  | S-DQ-3-2 | Negative | 24168 | 1.68 | 16.98 |
|  | S-DQ-3-3 | Negative | 23363 | 1.52 | 14.39 |
|  | S-QQ-3-1 | Negative | 6848 | 1.97 | 21.67 |
|  | S-QQ-3-2 | Negative | 10000 | 1.98 | 4.67 |
|  | S-QQ-3-3 | Negative | 8253 | 1.97 | 39 |
|  | S-Z-3-1 | Negative | 14365 | 1.73 | 18.65 |
|  | S-Z-3-2 | Negative | 15744 | 1.68 | 7.52 |
|  | S-Z-3-3 | Negative | 13872 | 1.76 | 19.82 |
| **Patient 4** | MN-4-1 | Negative | 9283 | 1.69 | 13.85 |
|  | MN-4-2 | Negative | 6941 | 1.13 | 8.19 |
|  | MN-4-3 | Negative | 7978 | 1.22 | 8.05 |
|  | DQ-4-1 | Negative | NA | 1.57 | 17.56 |
|  | DQ-4-2 | Negative | 14267 | 1.01 | 28.38 |
|  | DQ-4-3 | Negative | 25241 | 0.88 | 20.74 |
|  | QQ-4-1 | Negative | 25254 | 2.04 | 4 |
|  | QQ-4-2 | Negative | 22484 | 2.04 | 2.32 |
|  | QQ-4-3 | Negative | 25833 | 1.97 | 6.17 |
|  | Z-4-1 | Negative | 60000 | 1.2 | 0 |
|  | Z-4-2 | Negative | NA | 1.41 | 0 |
|  | Z-4-3 | Negative | 37105 | 1.3 | 0.28 |
|  | S-MN-4-1 | Negative | 19372 | 1.53 | 5.85 |
|  | S-MN-4-2 | Negative | 19963 | 1.68 | 4.35 |
|  | S-MN-4-3 | Negative | 25423 | 1.45 | 4.81 |
|  | S-DQ-4-1 | Negative | NA | 0.83 | 0.42 |
|  | S-DQ-4-2 | Negative | NA | 1.55 | 0.45 |
|  | S-DQ-4-3 | Negative | NA | 0.66 | 0.6 |
|  | S-QQ-4.2 | Negative | 29335 | 1.94 | 42.9 |
|  | S-QQ-4-1 | Negative | 24146 | 2.01 | 42.52 |
|  | S-QQ-4-3 | Negative | 32335 | 2.02 | 42.86 |
|  | S-Z-4-1 | Negative | 53432 | 1.34 | 89.13 |
|  | S-Z-4-2 | Negative | 51172 | 1.3 | 81.55 |
|  | S-Z-4-3 | Negative | 55846 | 1.49 | 56.9 |
| **Patient 5** | MN-5-1 | Negative | 11079 | 1.83 | 41.64 |
|  | MN-5-2 | Negative | 10779 | 1.83 | 44.08 |
|  | MN-5-3 | Negative | 10779 | 1.79 | 41.09 |
|  | DQ-5-1 | Negative | 7951 | 1.89 | 19.17 |
|  | DQ-5-2 | Negative | 9400 | 2.12 | 11.35 |
|  | DQ-5-3 | Negative | 8400 | 1.9 | 23.98 |
|  | QQ-5-1 | Negative | 9372 | 2.02 | 15.52 |
|  | QQ-5-2 | Negative | 10169 | 2.08 | 13.89 |
|  | QQ-5-3 | Negative | 8739 | 1.97 | 12.75 |
|  | Z-5-1 | Negative | 13000 | 1.64 | 41.31 |
|  | Z-5-2 | Negative | 15580 | 1.72 | 81.28 |
|  | Z-5-3 | Negative | 13232 | 1.7 | 42.6 |
|  | S-MN-5-1 | Negative | 21019 | 1.77 | 12 |
|  | S-MN-5-2 | Negative | 21756 | 1.67 | 11.86 |
|  | S-MN-5-3 | Negative | 23723 | 1.75 | 10.57 |
|  | S-DQ-5-1 | Negative | 20921 | 1.9 | 20.73 |
|  | S-DQ-5-2 | Negative | 20853 | 1.93 | 19.54 |
|  | S-DQ-5-3 | Negative | 18193 | 1.96 | 21.49 |
|  | S-QQ-5-1 | Negative | 22042 | 2.04 | 20.25 |
|  | S-QQ-5-2 | Negative | 18963 | 2.01 | 24.24 |
|  | S-QQ-5-3 | Negative | 20048 | 2.09 | 21.34 |
|  | S-Z-5-1 | Negative | 13694 | 1.55 | 25.42 |
|  | S-Z-5-2 | Negative | 14122 | 1.77 | 30.98 |
|  | S-Z-5-3 | Negative | 14213 | 1.75 | 28.96 |
| **Patient 6** | MN-6-1 | Positive | 11294 | 1.75 | 25.42 |
|  | MN-6-2 | Positive | 10183 | 1.81 | 27.57 |
|  | MN-6-3 | Positive | 10343 | 1.81 | 33.2 |
|  | DQ-6-1 | Positive | 14122 | 1.89 | 34.42 |
|  | DQ-6-2 | Positive | 13512 | 1.86 | 33.24 |
|  | DQ-6-3 | Positive | 14966 | 1.84 | 39.71 |
|  | QQ-6-1 | Positive | 11258 | 1.87 | 22.52 |
|  | QQ-6-2 | Positive | 10984 | 1.88 | 43.86 |
|  | QQ-6-3 | Positive | 11231 | 1.93 | 38.43 |
|  | Z-6-1 | Positive | 18570 | 1.49 | 4.9 |
|  | Z-6-2 | Positive | 15968 | 1.19 | 4.39 |
|  | Z-6-3 | Positive | 16398 | 1.48 | 4.13 |
|  | S-MN-6-1 | Positive | 23753 | 1.62 | 12.99 |
|  | S-MN-6-2 | Positive | NA | 1.7 | 11.78 |
|  | S-MN-6-3 | Positive | 11148 | 1.75 | 11.37 |
|  | S-DQ-6-1 | Positive | 17515 | 1.89 | 29.89 |
|  | S-DQ-6-2 | Positive | NA | 1.9 | 25.91 |
|  | S-DQ-6-3 | Positive | 17518 | 1.93 | 25.07 |
|  | S-QQ-6-1 | Positive | 15798 | 1.98 | 29.33 |
|  | S-QQ-6-2 | Positive | 16316 | 1.95 | 21.74 |
|  | S-QQ-6-3 | Positive | 17485 | 1.85 | 23.45 |
|  | S-Z-6-1 | Positive | 13546 | 1.75 | 26.55 |
|  | S-Z-6-2 | Positive | 15403 | 1.57 | 22.18 |
|  | S-Z-6-3 | Positive | 17485 | 1.46 | 17.87 |
| **Patient 7** | MN-7-1 | Positive | 12023 | 1.8 | 28.39 |
|  | MN-7-2 | Positive | 14636 | 1.85 | 22.61 |
|  | MN-7-3 | Positive | 12289 | 1.87 | 31.68 |
|  | DQ-7-1 | Positive | NA | 2.27 | 7.04 |
|  | DQ-7-2 | Positive | 20600 | 3.1 | 6.18 |
|  | DQ-7-3 | Positive | 21595 | 3.63 | 5.43 |
|  | QQ-7-1 | Positive | NA | 1.91 | 27.13 |
|  | QQ-7-2 | Positive | 12220 | 1.88 | 32.4 |
|  | QQ-7-3 | Positive | 10952 | 2.08 | 27.22 |
|  | Z-7-1 | Positive | 15403 | 1.68 | 59.54 |
|  | Z-7-2 | Positive | 16102 | 1.82 | 54.12 |
|  | Z-7-3 | Positive | 13727 | 1.65 | 46.61 |
|  | S-MN-7-1 | Positive | 28575 | 1.59 | 7.14 |
|  | S-MN-7-2 | Positive | 25642 | 1.86 | 11.37 |
|  | S-MN-7-3 | Positive | 29828 | 1.8 | 11.31 |
|  | S-DQ-7-1 | Positive | 24491 | 2.03 | 25.66 |
|  | S-DQ-7-2 | Positive | 28685 | 1.95 | 19.36 |
|  | S-DQ-7-3 | Positive | 24257 | 1.98 | 23.88 |
|  | S-QQ-7-1 | Positive | 24692 | 1.96 | 20.96 |
|  | S-QQ-7-2 | Positive | 24880 | 2.04 | 22.94 |
|  | S-QQ-7-3 | Positive | 20147 | 1.98 | 15.36 |
|  | S-Z-7-1 | Positive | 19129 | 1.7 | 42.51 |
|  | S-Z-7-2 | Positive | 20682 | 1.69 | 40.36 |
|  | S-Z-7-3 | Positive | 18020 | 1.61 | 25.45 |
| **Patient 8** | MN-8-1 | Negative | 12289 | 1.84 | 12.95 |
|  | MN-8-2 | Negative | 15536 | 1.77 | 13.96 |
|  | MN-8-3 | Negative | NA | 1.7 | 13.85 |
|  | DQ-8-1 | Negative | 12191 | 1.83 | 12.06 |
|  | DQ-8-2 | Negative | 14180 | 0.49 | 4.28 |
|  | DQ-8-3 | Negative | 15034 | 1.48 | 6.32 |
|  | QQ-8-1 | Negative | 13546 | 2.12 | 10.15 |
|  | QQ-8-2 | Negative | 14495 | 2.12 | 13.02 |
|  | QQ-8-3 | Negative | 12877 | 2.09 | 8.11 |
|  | Z-8-1 | Negative | NA | 1.4 | 9.56 |
|  | Z-8-2 | Negative | NA | 1.36 | 6.8 |
|  | Z-8-3 | Negative | NA | 1.44 | 10.11 |
|  | S-MN-8-1 | Negative | 16516 | 1.79 | 7 |
|  | S-MN-8-2 | Negative | 16114 | 1.54 | 8 |
|  | S-MN-8-3 | Negative | 19592 | 1.58 | 4.91 |
|  | S-DQ-8-1 | Negative | 10802 | 2.01 | 19.16 |
|  | S-DQ-8-2 | Negative | 10757 | 1.82 | 12.29 |
|  | S-DQ-8-3 | Negative | 11573 | 2.04 | 13.34 |
|  | S-QQ-8-1 | Negative | 12877 | 2.08 | 11.14 |
|  | S-QQ-8-2 | Negative | 12224 | 2.04 | 8.94 |
|  | S-QQ-8-3 | Negative | 12636 | 20.9 | 10.72 |
|  | S-Z-8-1 | Negative | 11635 | 1.4 | 12.12 |
|  | S-Z-8-2 | Negative | 12257 | 1.31 | 11.86 |
|  | S-Z-8-3 | Negative | 11860 | 1.44 | 8.64 |
| **Patient 9** | MN-9-1 | Negative | NA | 1.88 | 15.74 |
|  | MN-9-2 | Negative | 11908 | 1.79 | 13.14 |
|  | MN-9-3 | Negative | 13058 | 1.92 | 19.9 |
|  | DQ-9-1 | Negative | 16422 | 1.46 | 7.48 |
|  | DQ-9-2 | Negative | 15277 | 1.57 | 8.25 |
|  | DQ-9-3 | Negative | 18502 | 1.54 | 11.2 |
|  | QQ-9-1 | Negative | 14928 | 2.11 | 10.03 |
|  | QQ-9-2 | Negative | 13965 | 2.19 | 11.88 |
|  | QQ-9-3 | Negative | 15437 | 2.12 | 11.35 |
|  | Z-9-1 | Negative | 22314 | 1.66 | 24.76 |
|  | Z-9-2 | Negative | 26470 | 1.76 | 15.85 |
|  | Z-9-3 | Negative | 17352 | 1.8 | 22.92 |
|  | S-MN-9-1 | Negative | 15769 | 2.02 | 7.67 |
|  | S-MN-9-2 | Negative | 17022 | 2.12 | 2.67 |
|  | S-MN-9-3 | Negative | 21381 | 1.46 | 4.91 |
|  | S-DQ-9-1 | Negative | 17092 | 1.66 | 20.02 |
|  | S-DQ-9-2 | Negative | 15820 | 1.49 | 22.31 |
|  | S-DQ-9-3 | Negative | 15611 | 1.61 | 19.39 |
|  | S-QQ-9-1 | Negative | 13968 | 2.03 | 15.14 |
|  | S-QQ-9-2 | Negative | 13181 | 2.03 | 21.86 |
|  | S-QQ-9-3 | Negative | 13382 | 2 | 17.5 |
|  | S-Z-9-1 | Negative | 16932 | 1.47 | 17.42 |
|  | S-Z-9-2 | Negative | 21462 | 1.56 | 9.47 |
|  | S-Z-9-3 | Negative | 18291 | 1.52 | 18.03 |
| **Patient 10** | MN-10-1 | Positive | 12660 | 1.53 | 17.01 |
|  | MN-10-2 | Positive | 10202 | 1.79 | 24.18 |
|  | MN-10-3 | Positive | 10899 | 1.8 | 15.41 |
|  | DQ-10-1 | Positive | 20733 | 1.81 | 31.43 |
|  | DQ-10-2 | Positive | NA | 1.75 | 26.25 |
|  | QQ-10-1 | Positive | 14441 | 2.12 | 21.26 |
|  | QQ-10-2 | Positive | 13620 | 2.11 | 20.18 |
|  | QQ-10-3 | Positive | NA | 2.1 | 17.18 |
|  | Z-10-1 | Positive | 18102 | 1.29 | 9.07 |
|  | Z-10-2 | Positive | 19679 | 1.28 | 5.95 |
|  | S-MN-10-1 | Positive | 19366 | 1.57 | 5.82 |
|  | S-MN-10-2 | Positive | 19024 | 1.37 | 7 |
|  | S-MN-10-3 | Positive | 21751 | 1.5 | 6.86 |
|  | S-DQ-10-1 | Positive | 21381 | 1.9 | 22.47 |
|  | S-DQ-10-2 | Positive | 21722 | 1.83 | 24.82 |
|  | S-QQ-10-1 | Positive | 16771 | 2.07 | 37.85 |
|  | S-QQ-10-2 | Positive | 15500 | 2.07 | 55.29 |
|  | S-QQ-10-3 | Positive | 15820 | 2.05 | 41.14 |
|  | S-Z-10-1 | Positive | 22087 | 1.71 | 45.6 |
|  | S-Z-10-2 | Positive | 18961 | 1.67 | 48.94 |
| **Patient 11** | MN-11-1 | Positive | 11756 | 1.79 | 32.7 |
|  | MN-11-2 | Positive | 12645 | 1.86 | 50.48 |
|  | MN-11-3 | Positive | 13099 | NA | 56.23 |
|  | DQ-11-1 | Positive | 14091 | 1.78 | 24.14 |
|  | DQ-11-2 | Positive | 17088 | 1.89 | 18.36 |
|  | DQ-11-3 | Positive | 12832 | 1.82 | 59 |
|  | QQ-11-1 | Positive | 9943 | 1.82 | 41.96 |
|  | QQ-11-2 | Positive | 10306 | 1.86 | 50.92 |
|  | QQ-11-3 | Positive | 10802 | 1.96 | 22.65 |
|  | Z-11-1 | Positive | NA | 1.38 | 2.11 |
|  | Z-11-2 | Positive | 16867 | 1.34 | 3.11 |
|  | Z-11-3 | Positive | 14544 | 1.87 | 4.09 |
|  | S-MN-11-1 | Positive | 21501 | 1.5 | 6.03 |
|  | S-MN-11-2 | Positive | 18993 | 1.69 | 6.22 |
|  | S-MN-11-3 | Positive | 19917 | 1.79 | 7.25 |
|  | S-DQ-11-1 | Positive | 12738 | 1.79 | 20.62 |
|  | S-DQ-11-2 | Positive | NA | 1.76 | 35.41 |
|  | S-DQ-11-3 | Positive | 11633 | 1.79 | 39.72 |
|  | S-QQ-11-1 | Positive | 16543 | 2.08 | 18.08 |
|  | S-QQ-11-2 | Positive | 15074 | 2.04 | 21.48 |
|  | S-QQ-11-3 | Positive | 16074 | 1.99 | 22.33 |
|  | S-Z-11-1 | Positive | 12207 | 1.65 | 26.94 |
|  | S-Z-11-2 | Positive | 13340 | 1.67 | 16.45 |
|  | S-Z-11-3 | Positive | 13866 | 1.71 | 25.51 |
| **Patient 13** | MN-13-1 | Negative | 12300 | 1.84 | 48.27 |
|  | MN-13-2 | Negative | 10457 | 1.9 | 31.93 |
|  | MN-13-3 | Negative | 10651 | 1.85 | 28.18 |
|  | DQ-13-1 | Negative | 10626 | 1.73 | 58.34 |
|  | DQ-13-2 | Negative | 10662 | 1.77 | 53.29 |
|  | DQ-13-3 | Negative | 13124 | 1.72 | 62.79 |
|  | QQ-13-1 | Negative | 10403 | 2.04 | 20.21 |
|  | QQ-13-2 | Negative | 10919 | 2.06 | 20.45 |
|  | QQ-13-3 | Negative | 10890 | 2.03 | 19.74 |
|  | Z-13-1 | Negative | 17042 | 1 | 0.3 |
|  | Z-13-2 | Negative | 18773 | 1.47 | 6.11 |
|  | Z-13-3 | Negative | 18198 | 1.21 | 16.4 |
|  | S-MN-13-1 | Negative | NA | 3.21 | 1.77 |
|  | S-MN-13-2 | Negative | 18899 | 0.4 | 5.54 |
|  | S-MN-13-3 | Negative | 17519 | 3.06 | 2.71 |
|  | S-DQ-13-1 | Negative | 11766 | 1.81 | 20.19 |
|  | S-DQ-13-2 | Negative | 16707 | 1.35 | 14.3 |
|  | S-DQ-13-3 | Negative | 12642 | 1.61 | 6.7 |
|  | S-QQ-13-1 | Negative | 18489 | 1.98 | 14.5 |
|  | S-QQ-13-2 | Negative | 13845 | 2.06 | 9.18 |
|  | S-QQ-13-3 | Negative | 17759 | 1.99 | 13.16 |
|  | S-Z-13-1 | Negative | NA | 1.17 | 8.31 |
|  | S-Z-13-2 | Negative | 14726 | 1.48 | 12.41 |
|  | S-Z-13-3 | Negative | 20478 | 1.16 | 11.77 |
| **Patient 14** | MN-14-1 | Negative | 12116 | 1.79 | 51.22 |
|  | MN-14-2 | Negative | 7078 | 1.74 | 50.3 |
|  | MN-14-3 | Negative | 7863 | 1.76 | 52.13 |
|  | DQ-14-1 | Negative | 13811 | 1.86 | 48.35 |
|  | DQ-14-2 | Negative | 16453 | 1.86 | 52.99 |
|  | DQ-14-3 | Negative | 12674 | 1.84 | 71.32 |
|  | QQ-14-1 | Negative | 10104 | 2.15 | 16.02 |
|  | QQ-14-2 | Negative | 12292 | 2.18 | 13.48 |
|  | QQ-14-3 | Negative | 12336 | 2.12 | 26.91 |
|  | Z-14-1 | Negative | 10744 | 1.7 | 134.84 |
|  | Z-14-2 | Negative | 11938 | 1.7 | 98.26 |
|  | Z-14-3 | Negative | 10714 | 1.55 | 113.43 |
|  | S-MN-14-1 | Negative | 26516 | 4.45 | 7 |
|  | S-MN-14-2 | Negative | 25529 | -3.97 | 4.76 |
|  | S-MN-14-3 | Negative | NA | 1.71 | 5.95 |
|  | S-DQ-14-1 | Negative | 23208 | 1.81 | 41.6 |
|  | S-DQ-14-2 | Negative | 26558 | 1.93 | 31.9 |
|  | S-DQ-14-3 | Negative | NA | 1.82 | 61.78 |
|  | S-QQ-14-1 | Negative | 19702 | 2.02 | 49.42 |
|  | S-QQ-14-2 | Negative | 16042 | 2.08 | 43.14 |
|  | S-QQ-14-3 | Negative | NA | 2.12 | 50.37 |
|  | S-Z-14-1 | Negative | 17588 | 1.5 | 61.56 |
|  | S-Z-14-2 | Negative | 17840 | 1.67 | 58.98 |
|  | S-Z-14-3 | Negative | 16409 | 1.49 | 93.11 |
| **Patient 15** | MN-15-1 | Negative | 9724 | 1.83 | 70.69 |
|  | MN-15-2 | Negative | 9065 | 1.49 | 23.08 |
|  | MN-15-3 | Negative | 11447 | 1.6 | 22.46 |
|  | DQ-15-1 | Negative | 25256 | 1.82 | 90.26 |
|  | DQ-15-2 | Negative | 22328 | 1.87 | 112.18 |
|  | DQ-15-3 | Negative | 22268 | 1.82 | 111.06 |
|  | QQ-15-1 | Negative | 11157 | 2.01 | 40 |
|  | QQ-15-2 | Negative | 13242 | 1.96 | 50.38 |
|  | QQ-15-3 | Negative | 15539 | 1.96 | 10.65 |
|  | Z-15-1 | Negative | 26991 | 1.8 | 220.96 |
|  | Z-15-2 | Negative | NA | 1.8 | 254.99 |
|  | Z-15-3 | Negative | 43056 | 1.72 | 233.22 |
|  | S-MN-15-1 | Negative | 24012 | 0.57 | 4.46 |
|  | S-MN-15-2 | Negative | 31535 | -3.95 | 4.75 |
|  | S-MN-15-3 | Negative | 50000 | 0.31 | 4.4 |
|  | S-DQ-15-1 | Negative | 19501 | 1.82 | 23.58 |
|  | S-DQ-15-2 | Negative | 19595 | 1.93 | 22.52 |
|  | S-DQ-15-3 | Negative | 16897 | 1.86 | 44.98 |
|  | S-QQ-15-1 | Negative | 18739 | 1.88 | 95.99 |
|  | S-QQ-15-2 | Negative | 17512 | 1.386 | 169.31 |
|  | S-QQ-15-3 | Negative | 16690 | 1.85 | 192.45 |
|  | S-Z-15-1 | Negative | 21711 | 1.78 | 225.9 |
|  | S-Z-15-2 | Negative | 22113 | 1.76 | 147.85 |
|  | S-Z-15-3 | Negative | 24462 | 1.78 | 214.71 |
| **Patient 16** | MN-16-1 | Positive | 12678 | 1.8 | 5.77 |
|  | MN-16-2 | Positive | 11483 | 2.19 | 3.69 |
|  | MN-16-3 | Positive | 11360 | 1.44 | 4.47 |
|  | DQ-16-1 | Positive | 28493 | 1.73 | 1.69 |
|  | DQ-16-2 | Positive | 37789 | 1.28 | 1.45 |
|  | DQ-16-3 | Positive | 25229 | 2.16 | 1.22 |
|  | QQ-16-1 | Positive | 54068 | 1.92 | 1.20 |
|  | QQ-16-2 | Positive | 52374 | 1.68 | 0.80 |
|  | QQ-16-3 | Positive | NA | 2.52 | 0.93 |
|  | Z-16-1 | Positive | 48807 | 1.42 | 3.22 |
|  | Z-16-2 | Positive | 24256 | 1.26 | 2.89 |
|  | Z-16-3 | Positive | 49726 | 1.61 | 2.88 |
|  | S-MN-16-1 | Positive | 20903 | 28.96 | 0.50 |
|  | S-MN-16-2 | Positive | 13833 | 1.11 | 0.55 |
|  | S-MN-16-3 | Positive | NA | 1.04 | 0.54 |
|  | S-DQ-16-1 | Positive | 24916 | 1.72 | 5.10 |
|  | S-DQ-16-2 | Positive | 32633 | 1.89 | 5.79 |
|  | S-DQ-16-3 | Positive | 21144 | 1.73 | 4.23 |
|  | S-QQ-16-1 | Positive | 21083 | 1.78 | 4.25 |
|  | S-QQ-16-2 | Positive | 19184 | 2.37 | 3.50 |
|  | S-QQ-16-3 | Positive | 21022 | 1.71 | 2.60 |
|  | S-Z-16-1 | Positive | 18737 | 1.37 | 6.08 |
|  | S-Z-16-2 | Positive | 21893 | 1.48 | 4.23 |
|  | S-Z-16-3 | Positive | 20744 | 1.43 | 4.52 |
| **Patient 17** | MN-17-1 | Positive | 13746 | 184 | 41.70 |
|  | MN-17-2 | Positive | 11891 | 1.91 | 27.82 |
|  | MN-17-3 | Positive | 12113 | 1.89 | 49.20 |
|  | DQ-17-1 | Positive | 20402 | 1.84 | 28.11 |
|  | DQ-17-2 | Positive | 23964 | 1.83 | 16.32 |
|  | DQ-17-3 | Positive | 16162 | 1.85 | 22.05 |
|  | QQ-17-1 | Positive | 18968 | 1.95 | 10.39 |
|  | QQ-17-2 | Positive | 19881 | 1.85 | 8.10 |
|  | QQ-17-3 | Positive | 20605 | 1.89 | 11.72 |
|  | Z-17-1 | Positive | 24515 | 1.6 | 93.60 |
|  | Z-17-2 | Positive | 22369 | 1.67 | 101.80 |
|  | Z-17-3 | Positive | 22752 | 1.75 | 97.80 |
|  | S-MN-17-1 | Positive | NA | 1.92 | 7.84 |
|  | S-MN-17-2 | Positive | 22954 | 1.89 | 5.79 |
|  | S-MN-17-3 | Positive | 23434 | 2.15 | 5.04 |
|  | S-DQ-17-1 | Positive | 18880 | 1.86 | 27.92 |
|  | S-DQ-17-2 | Positive | 17887 | 1.86 | 36.89 |
|  | S-DQ-17-3 | Positive | 21946 | 1.87 | 27.23 |
|  | S-QQ-17-1 | Positive | 19718 | 1.93 | 20.96 |
|  | S-QQ-17-2 | Positive | 19749 | 1.87 | 27.90 |
|  | S-QQ-17-3 | Positive | 22750 | 1.74 | 25.36 |
|  | S-Z-17-1 | Positive | 20347 | 1.69 | 54.60 |
|  | S-Z-17-2 | Positive | 21379 | 1.71 | 47.52 |
|  | S-Z-17-3 | Positive | 21621 | 1.72 | 41.38 |
| **Patient 18** | MN-18-1 | Positive | 12407 | -3.61 | 7.86 |
|  | MN-18-2 | Positive | 11922 | 1.52 | 5.02 |
|  | MN-18-3 | Positive | 14586 | 1.66 | 6.07 |
|  | DQ-18-1 | Positive | 22034 | 1.11 | 2.36 |
|  | DQ-18-2 | Positive | 24975 | 0.63 | 1.84 |
|  | DQ-18-3 | Positive | 21507 | 0.86 | 1.26 |
|  | QQ-18-1 | Positive | 14329 | 2.32 | 4.19 |
|  | QQ-18-2 | Positive | 13523 | 2.19 | 6.43 |
|  | QQ-18-3 | Positive | 20990 | 4.15 | 1.79 |
|  | Z-18-1 | Positive | 31132 | 1.31 | 5.47 |
|  | Z-18-2 | Positive | NA | 1.62 | 4.70 |
|  | Z-18-3 | Positive | 29167 | 1.36 | 5.47 |
|  | S-MN-18-1 | Positive | 19974 | 1.4 | 2.85 |
|  | S-MN-18-2 | Positive | 19881 | 2.6 | 3.57 |
|  | S-MN-18-3 | Positive | 18911 | 3.23 | 3.79 |
|  | S-DQ-18-1 | Positive | 16863 | -54.14 | 9.79 |
|  | S-DQ-18-2 | Positive | 12283 | 0.19 | 12.96 |
|  | S-DQ-18-3 | Positive | NA | 0.51 | 18.21 |
|  | S-QQ-18-1 | Positive | 18534 | 2.68 | 3.28 |
|  | S-QQ-18-2 | Positive | 19605 | 2.21 | 2.97 |
|  | S-QQ-18-3 | Positive | NA | 2.35 | 2.46 |
|  | S-Z-18-1 | Positive | 27883 | 1.63 | 9.96 |
|  | S-Z-18-2 | Positive | 23580 | 1.42 | 13.32 |
|  | S-Z-18-3 | Positive | NA | 1.49 | 15.31 |
| **Patient 19** | MN-19-1 | Positive | 24137 | 0.97 | 3.59 |
|  | MN-19-2 | Positive | NA | 0.98 | 3.29 |
|  | MN-19-3 | Positive | 21204 | 1.47 | 3.06 |
|  | DQ-19-1 | Positive | 29356 | 1.91 | 4.46 |
|  | DQ-19-2 | Positive | 52476 | 2.21 | 4.85 |
|  | DQ-19-3 | Positive | 53551 | 1.27 | 3.84 |
|  | QQ-19-1 | Positive | NA | 1.43 | 1.57 |
|  | QQ-19-2 | Positive | NA | 0.64 | 1.82 |
|  | QQ-19-3 | Positive | NA | 0.99 | 1.14 |
|  | Z-19-1 | Positive | NA | 1.17 | 1.38 |
|  | Z-19-2 | Positive | NA | 1.13 | 0.98 |
|  | Z-19-3 | Positive | NA | 1.56 | 1.43 |
|  | S-MN-19-1 | Positive | 23289 | 1.52 | 4.69 |
|  | S-MN-19-2 | Positive | 27808 | 1.59 | 6.55 |
|  | S-MN-19-3 | Positive | 24709 | 1.35 | 3.04 |
|  | S-DQ-19-1 | Positive | 26338 | 1.66 | 9.60 |
|  | S-DQ-19-2 | Positive | 27703 | 1.85 | 13.62 |
|  | S-DQ-19-3 | Positive | 22093 | 1.64 | 17.79 |
|  | S-QQ-19-1 | Positive | 22318 | 2.15 | 17.19 |
|  | S-QQ-19-2 | Positive | 22838 | 2.03 | 21.03 |
|  | S-QQ-19-3 | Positive | 20697 | 2.08 | 11.86 |
|  | S-Z-19-1 | Positive | 23156 | 1.62 | 41.04 |
|  | S-Z-19-2 | Positive | 25586 | 1.66 | 38.93 |
|  | S-Z-19-3 | Positive | 23124 | 1.68 | 2.07 |

**Supplementary Table 2. Estimation of the Firmicutes/Bacteroidetes ratio. This ratio was calculated for every individual extracted by the eight different extraction protocols using the SMS data.**

**Supplementary Table 3. Differential abundance of taxa between standard vs SPD-combined protocols.**

|  |  | **baseMean** | **log2FoldChange** | **padj** |
| --- | --- | --- | --- | --- |
| **Patient 1** | Acholeplasmataceae | 1751 | 2.63 | 4.91E-11 |
|  | Akkermansiaceae | 275 | -2.25 | 7.05E-05 |
|  | Barnesiellaceae | 1452 | 1.07 | 0.00483918 |
|  | Bifidobacteriaceae | 1845 | -2.21 | 1.65E-12 |
|  | Butyricicoccaceae | 257 | -1.71 | 0.00231287 |
|  | Clostridia_vadinBB60_group | 333 | 4.50 | 5.79E-12 |
|  | Coriobacteriaceae | 915 | -1.45 | 0.01296127 |
|  | Erysipelotrichaceae | 2780 | -3.36 | 1.87E-14 |
|  | Gastranaerophilales | 785 | 1.81 | 2.31E-10 |
|  | Izemoplasmatales | 190 | 2.80 | 1.31E-06 |
|  | Lactobacillaceae | 162 | -3.60 | 0.00027352 |
|  | Oscillospiraceae | 3698 | 1.11 | 1.74E-05 |
|  | Peptostreptococcaceae | 56 | -7.64 | 1.89E-15 |
|  | Streptococcaceae | 292 | -1.61 | 0.0197725 |
|  | Sutterellaceae | 2258 | 1.81 | 9.74E-13 |
|  | UCG-010 | 1105 | 1.66 | 5.87E-12 |
|  | uncultured | 1109 | 1.68 | 1.06E-10 |
| **Patient 2** | Actinomycetaceae | 3743 | 2.11 | 0.00744285 |
|  | Aerococcaceae | 358 | 2.46 | 0.03125459 |
|  | Alicyclobacillaceae | 101 | -4.29 | 7.10E-05 |
|  | Bacteroidaceae | 960 | -3.09 | 1.01E-06 |
|  | Bifidobacteriaceae | 2138 | 1.15 | 0.00043488 |
|  | Clostridiaceae | 4821 | 1.55 | 5.55E-06 |
|  | Enterococcaceae | 7636 | 2.02 | 1.60E-07 |
|  | Eubacteriaceae | 173 | 2.65 | 0.00688895 |
|  | Marinifilaceae | 109 | -4.52 | 7.21E-05 |
|  | Monoglobaceae | 232 | -2.11 | 0.0159544 |
|  | Oscillospiraceae | 5044 | 1.48 | 7.10E-05 |
|  | Peptostreptococcaceae | 4537 | 2.02 | 2.12E-12 |
|  | Porphyromonadaceae | 916 | -1.29 | 0.00065451 |
|  | Rhizobiaceae | 3505 | -1.95 | 5.86E-15 |
|  | Rikenellaceae | 424 | -2.90 | 0.00065451 |
|  | Streptococcaceae | 3428 | 1.83 | 1.81E-05 |
|  | Tannerellaceae | 2478 | -2.35 | 3.79E-14 |
| **Patient 3** | Acidaminococcaceae | 904 | 1.21 | 0.02678983 |
|  | Bifidobacteriaceae | 3550 | 1.43 | 3.36E-08 |
|  | Butyricicoccaceae | 1568 | -3.51 | 4.67E-07 |
|  | Clostridiaceae | 430 | 1.59 | 0.00055517 |
|  | Coriobacteriaceae | 6807 | 1.48 | 2.87E-07 |
|  | Coriobacteriales_Incertae_Sedis | 928 | 1.44 | 0.00068567 |
|  | Erysipelotrichaceae | 168 | 1.28 | 0.01811718 |
|  | Eubacteriaceae | 65 | 3.01 | 0.00228759 |
|  | Lactobacillaceae | 5387 | 1.09 | 0.00011776 |
|  | Marinifilaceae | 646 | -1.83 | 2.10E-06 |
|  | Pasteurellaceae | 145 | -4.76 | 4.67E-07 |
|  | Peptostreptococcaceae | 1382 | 2.25 | 7.36E-11 |
|  | Rhizobiaceae | 1213 | -1.08 | 0.02441111 |
|  | Rikenellaceae | 5421 | -1.51 | 5.29E-07 |
|  | Streptococcaceae | 8634 | 2.05 | 4.10E-10 |
|  | Sutterellaceae | 1127 | -1.53 | 0.0001798 |
|  | uncultured | 614 | -2.06 | 4.10E-10 |
|  | Victivallaceae | 44 | -4.89 | 2.52E-05 |
| **Patient 4** | Alicyclobacillaceae | 107 | -3.66 | 2.58E-05 |
|  | Bacteroidaceae | 18377 | -1.74 | 7.46E-10 |
|  | Barnesiellaceae | 2237 | -3.78 | 1.36E-10 |
|  | Clostridiaceae | 6208 | 1.81 | 2.35E-07 |
|  | Eggerthellaceae | 2259 | 1.19 | 0.04809233 |
|  | Eubacteriaceae | 233 | 1.38 | 0.03677796 |
|  | Lactobacillaceae | 17 | 4.67 | 0.00285218 |
|  | Peptostreptococcaceae | 5657 | 2.11 | 5.72E-08 |
|  | Rhizobiaceae | 3805 | -1.64 | 5.27E-09 |
|  | Rikenellaceae | 5807 | -2.45 | 2.79E-16 |
|  | Saccharimonadales | 223 | -2.60 | 0.00195037 |
|  | Streptococcaceae | 388 | 2.12 | 0.00143793 |
|  | Tannerellaceae | 2547 | -2.36 | 2.60E-14 |
|  | UCG-010 | 759 | -3.33 | 6.69E-07 |
| **Patient 5** | Bifidobacteriaceae | 26125 | 1.59 | 6.79E-19 |
|  | Coriobacteriaceae | 257 | 2.39 | 0.01468035 |
|  | Eggerthellaceae | 1314 | 1.65 | 6.01E-09 |
|  | Enterococcaceae | 1167 | 2.24 | 0.00058382 |
|  | Erysipelatoclostridiaceae | 6210 | 1.16 | 3.56E-08 |
|  | Gemellaceae | 14 | 4.17 | 0.0103562 |
|  | Lactobacillaceae | 147 | 4.02 | 0.0005361 |
|  | Pasteurellaceae | 104 | -4.82 | 3.59E-07 |
|  | Ruminococcaceae | 7489 | -4.25 | 1.04E-290 |
|  | Streptococcaceae | 693 | 2.32 | 1.86E-19 |
|  | Sutterellaceae | 3870 | -2.46 | 2.40E-08 |
|  | Veillonellaceae | 2106 | -1.02 | 0.00826155 |
| **Patient 6** | Barnesiellaceae | 3127 | -2.38 | 2.11E-17 |
|  | Bifidobacteriaceae | 162 | -1.74 | 0.02992546 |
|  | Christensenellaceae | 309 | 1.07 | 2.19E-05 |
|  | Erysipelotrichaceae | 5309 | 1.50 | 1.16E-16 |
|  | Flavobacteriaceae | 392 | -1.43 | 4.06E-07 |
|  | Leuconostocaceae | 25 | 3.11 | 0.00129652 |
|  | Marinifilaceae | 1309 | -2.38 | 3.25E-14 |
|  | Peptostreptococcaceae | 3355 | 2.14 | 5.74E-10 |
|  | Prevotellaceae | 322 | -1.09 | 6.33E-06 |
|  | Rhizobiaceae | 2553 | -1.02 | 1.68E-05 |
|  | Rikenellaceae | 17073 | -1.42 | 8.40E-09 |
|  | Streptococcaceae | 2206 | 2.18 | 2.03E-10 |
| **Patient 7** | Actinomycetaceae | 127 | 5.26 | 5.57E-17 |
|  | Bifidobacteriaceae | 14 | 4.10 | 5.69E-05 |
|  | Eggerthellaceae | 887 | 2.19 | 6.87E-47 |
|  | Enterococcaceae | 5008 | 2.56 | 8.32E-16 |
|  | Erysipelatoclostridiaceae | 2242 | 1.25 | 6.36E-14 |
|  | Erysipelotrichaceae | 990 | 1.03 | 1.28E-07 |
|  | Lactobacillaceae | 468 | 2.69 | 1.16E-12 |
|  | Oscillospiraceae | 937 | -2.33 | 8.59E-16 |
|  | Peptostreptococcales-Tissierellales | 43 | 5.04 | 2.32E-12 |
|  | Rikenellaceae | 6804 | -1.72 | 0.00654832 |
|  | Streptococcaceae | 5196 | 2.60 | 1.29E-30 |
| **Patient 8** | [Eubacterium]_coprostanoligenes_group | 49 | -4.08 | 0.02429421 |
|  | Alicyclobacillaceae | 149 | -4.48 | 5.84E-05 |
|  | Anaerovoracaceae | 7 | -3.98 | 0.04612696 |
|  | Bacteroidaceae | 14672 | -1.14 | 0.00041179 |
|  | Barnesiellaceae | 21 | -3.19 | 0.04063983 |
|  | Campylobacteraceae | 10 | -4.11 | 0.04063983 |
|  | Desulfovibrionaceae | 154 | -1.55 | 0.03285966 |
|  | Enterobacteriaceae | 2188 | -1.43 | 1.01E-07 |
|  | Pasteurellaceae | 632 | -2.45 | 0.00021247 |
|  | Rhizobiaceae | 3431 | -2.41 | 4.69E-20 |
|  | Rikenellaceae | 463 | -4.05 | 0.00041179 |
|  | Selenomonadaceae | 3170 | -3.80 | 5.55E-09 |
|  | Sutterellaceae | 1783 | -1.67 | 0.00593219 |
|  | Tannerellaceae | 2573 | -1.12 | 0.00144093 |
| **Patient 9** | [Eubacterium]_coprostanoligenes_group | 658 | -2.89 | 1.23E-06 |
|  | Alicyclobacillaceae | 100 | -4.95 | 3.25E-06 |
|  | Bacteroidaceae | 11379 | -1.89 | 7.65E-14 |
|  | Bifidobacteriaceae | 5569 | 1.21 | 1.92E-12 |
|  | Coriobacteriales_Incertae_Sedis | 457 | 1.48 | 0.02726016 |
|  | Lactobacillaceae | 5056 | 1.00 | 1.85E-06 |
|  | Oscillospiraceae | 22 | -6.56 | 0.00052118 |
|  | Peptostreptococcaceae | 95 | 3.33 | 0.01422513 |
|  | Rhizobiaceae | 2990 | -1.65 | 2.35E-21 |
|  | Rikenellaceae | 93 | -5.54 | 3.27E-07 |
|  | Streptococcaceae | 6852 | 1.21 | 6.27E-10 |
|  | Sutterellaceae | 116 | -9.30 | 1.13E-14 |
|  | Tannerellaceae | 2480 | -1.70 | 6.77E-12 |
| **Patient 10** | Atopobiaceae | 143 | 1.41 | 0.00569791 |
|  | Bacteroidaceae | 5503 | -1.18 | 2.13E-08 |
|  | Bifidobacteriaceae | 242 | 1.86 | 0.01291712 |
|  | Enterococcaceae | 562 | 1.59 | 0.02637514 |
|  | Gemellaceae | 29 | 3.32 | 0.0008464 |
|  | Lactobacillaceae | 2950 | 1.22 | 2.46E-07 |
|  | Marinifilaceae | 741 | -3.79 | 1.60E-14 |
|  | Rhizobiaceae | 863 | -1.43 | 8.53E-07 |
|  | Rikenellaceae | 3459 | -2.62 | 1.60E-14 |
|  | Staphylococcaceae | 38 | 6.50 | 1.82E-13 |
|  | Streptococcaceae | 3858 | 2.30 | 6.40E-15 |
| **Patient 11** | Atopobiaceae | 703 | 1.31 | 0.01032524 |
|  | Barnesiellaceae | 2771 | -1.42 | 8.06E-11 |
|  | Eggerthellaceae | 440 | 1.46 | 0.01727874 |
|  | Marinifilaceae6 | 1200 | -1.34 | 7.82E-07 |
|  | Streptococcaceae | 171 | 1.54 | 0.04800077 |
| **Patient 13** | Alicyclobacillaceae | 74 | -2.07 | 0.00913102 |
|  | Marinifilaceae | 372 | -2.60 | 1.22E-05 |
|  | Peptostreptococcaceae | 8852 | 1.62 | 4.37E-21 |
|  | Rhizobiaceae | 2019 | -1.26 | 0.00841998 |
|  | Rikenellaceae | 2568 | -1.44 | 1.48E-08 |
|  | Streptococcaceae | 7799 | 1.85 | 2.50E-13 |
| **Patient 14** | Actinomycetaceae | 190 | 1.79 | 0.04157915 |
|  | Bifidobacteriaceae | 10882 | 1.67 | 2.73E-13 |
|  | Eggerthellaceae | 700 | 1.83 | 0.00010116 |
|  | Lactobacillaceae | 1263 | 2.09 | 4.33E-13 |
|  | Marinifilaceae | 1677 | -1.94 | 3.27E-16 |
|  | Pasteurellaceae | 94 | -2.88 | 0.00586775 |
|  | Prevotellaceae | 460 | -1.23 | 0.02433728 |
|  | Ruminococcaceae | 599 | -2.84 | 0.00036888 |
|  | Streptococcaceae | 15680 | 1.88 | 1.04E-14 |
| **Patient 15** | [Eubacterium]_coprostanoligenes_group | 990 | -2.49 | 2.60E-21 |
|  | Acidaminococcaceae | 1954 | -1.40 | 7.03E-17 |
|  | Bifidobacteriaceae | 447 | 1.21 | 7.16E-08 |
|  | Clostridia_UCG-014 | 27 | -2.78 | 0.00293747 |
|  | Clostridiaceae | 84 | 1.68 | 0.02028067 |
|  | Coriobacteriaceae | 1017 | 1.36 | 8.19E-10 |
|  | Lachnospiraceae | 3567 | 1.35 | 7.03E-17 |
|  | Marinifilaceae | 145 | -4.21 | 4.60E-07 |
|  | Peptostreptococcaceae | 134 | 1.89 | 0.00640291 |
|  | Rhizobiaceae | 175 | -1.73 | 0.00538586 |
|  | Rikenellaceae | 532 | -1.50 | 9.69E-07 |
|  | Streptococcaceae | 1059 | 2.00 | 4.86E-09 |
|  | uncultured | 48 | -6.46 | 1.85E-15 |
| **Patient 16** | Alicyclobacillaceae | 804 | -3.27 | 2.80E-15 |
|  | Peptostreptococcaceae | 6423 | -1.72 | 1.11E-14 |
|  | Rhizobiaceae | 16382 | -1.77 | 5.32E-22 |
|  | Streptococcaceae | 9733 | 1.85 | 9.06E-13 |
| **Patient 17** | Alicyclobacillaceae | 67 | -3.07 | 2.67E-06 |
|  | Clostridiaceae | 6063 | -1.90 | 1.99E-64 |
|  | Enterococcaceae | 1156 | 2.52 | 9.07E-13 |
|  | Erysipelatoclostridiaceae | 811 | 2.51 | 1.33E-29 |
|  | Gemellaceae | 65 | 1.74 | 0.01940245 |
|  | Lachnospiraceae | 14116 | 1.13 | 2.11E-27 |
|  | Streptococcaceae | 324 | 1.46 | 0.02598559 |
|  | Sutterellaceae | 2546 | -1.00 | 3.25E-10 |
|  | Veillonellaceae | 156 | -2.18 | 0.00191278 |
| **Patient 18** | Alicyclobacillaceae | 215 | -3.12 | 4.76E-05 |
|  | Bacteroidaceae | 993 | -1.76 | 4.60E-10 |
|  | Enterobacteriaceae | 1983 | -1.05 | 1.10E-07 |
|  | Hafniaceae | 14144 | -1.40 | 2.18E-09 |
|  | Rhizobiaceae | 7247 | -2.63 | 4.95E-44 |
|  | Streptococcaceae | 12148 | 1.27 | 5.74E-16 |
|  | Tannerellaceae | 187 | -2.74 | 0.0007075 |
|  | Veillonellaceae | 985 | -1.15 | 1.57E-06 |
| **Patient 19** | Actinomycetaceae | 91 | 3.04 | 0.00019983 |
|  | Alicyclobacillaceae | 117 | -2.65 | 0.00011616 |
|  | Bifidobacteriaceae | 177 | 2.25 | 0.00038716 |
|  | Clostridia_vadinBB60_group | 45 | -6.54 | 2.67E-14 |
|  | Clostridiaceae | 418 | 2.32 | 1.96E-08 |
|  | Enterococcaceae | 4599 | 1.33 | 2.67E-14 |
|  | Erysipelotrichaceae | 358 | 1.50 | 0.00395588 |
|  | Fusobacteriaceae | 1034 | -2.77 | 3.03E-44 |
|  | Lachnospiraceae | 602 | 1.46 | 5.53E-09 |
|  | Pasteurellaceae | 132 | -3.61 | 2.31E-05 |
|  | Peptostreptococcaceae | 1072 | 2.54 | 4.36E-19 |
|  | Prevotellaceae | 119 | -1.75 | 0.02329952 |
|  | Rhizobiaceae | 3112 | -1.67 | 4.19E-21 |
|  | Ruminococcaceae | 1762 | -1.42 | 1.04E-15 |
|  | Streptococcaceae | 2629 | 1.66 | 2.65E-11 |
|  | Sutterellaceae | 220 | -4.24 | 4.95E-08 |
|  | Veillonellaceae | 4768 | -1.30 | 1.96E-32 |
